# Gender Agreement Attraction in Russian: Production and Comprehension Evidence

**DOI:** 10.3389/fpsyg.2016.01651

**Published:** 2016-11-04

**Authors:** Natalia Slioussar, Anton Malko

**Affiliations:** ^1^School of Linguistics, Higher School of EconomicsMoscow, Russia; ^2^Faculty of Liberal Arts and Sciences, Saint-Petersburg State UniversitySaint-Petersburg, Russia; ^3^Department of Linguistics, University of MarylandCollege Park, MD, USA

**Keywords:** agreement, gender, attraction, production, comprehension, Russian

## Abstract

Agreement attraction errors (such as the number error in the example “The key to the cabinets are rusty”) have been the object of many studies in the last 20 years. So far, almost all production experiments and all comprehension experiments looked at binary features (primarily at number in Germanic, Romance, and some other languages, in several cases at gender in Romance languages). Among other things, it was noted that both in production and in comprehension, attraction effects are much stronger for some feature combinations than for the others: they can be observed in the sentences with singular heads and plural dependent nouns (e.g.,“The key to the cabinets…”), but not in the sentences with plural heads and singular dependent nouns (e.g., “The keys to the cabinet…”). Almost all proposed explanations of this asymmetry appeal to feature markedness, but existing findings do not allow teasing different approaches to markedness apart. We report the results of four experiments (one on production and three on comprehension) studying subject-verb gender agreement in Russian, a language with three genders. Firstly, we found attraction effects both in production and in comprehension, but, unlike in the case of number agreement, they were not parallel (in production, feminine gender triggered strongest effects, while neuter triggered weakest effects, while in comprehension, masculine triggered weakest effects). Secondly, in the comprehension experiments attraction was observed for all dependent noun genders, but only for a subset of head noun genders. This goes against the traditional assumption that the features of the dependent noun are crucial for attraction, showing the features of the head are more important. We demonstrate that this approach can be extended to previous findings on attraction and that there exists other evidence for it. In total, these findings let us reconsider the question which properties of features are crucial for agreement attraction in production and in comprehension.

## 1. Introduction

### 1.1. The phenomenon of agreement attraction

Grammatical agreement is one of the most basic linguistic operations. It is well-known, however, that it is not always accurate. In the last 20 years many studies have looked at so-called agreement attraction errors, exemplified in (1). In (1a) the verb agrees not with the head of the subject NP *key*^1^, but with another, embedded NP *cabinets* (we will further call such NPs “attractors”). In (1b) the verb in a relative clause agrees with the subject of the matrix clause.

(1)
  - The key to the cabinets **were** rusty (Bock and Miller, 1991).
  - The musicians who the reviewer **praise** so highly will probably win a Grammy (Wagers et al., 2009).

Agreement attraction errors are observed in spontaneous speech and in well-edited texts. They have also been studied experimentally, mostly in English, but also in French, Spanish, Italian, Dutch, German, and some other languages (Bock and Miller, 1991; Vigliocco et al., 1995, 1996; Pearlmutter et al., 1999; Anton-Mendez et al., 2002; Hartsuiker et al., 2003, to name just a few). The first accounts suggested that the verb simply agrees with the linearly closest noun (Jespersen, 1924; Quirk et al., 1972; Francis, 1986, a.o.). However, later studies demonstrated that agreement attraction is a structural phenomenon. For example, Vigliocco and Nicol (1998) showed that people make attraction errors producing questions, e.g., “Are the helicopter for the flights safe?.” Various factors that influence attraction have also been identified. However, the overwhelming majority of studies focused on number agreement in the languages where number has only two values: singular and plural. It is not clear to what extent these results can be generalized to other cases.

In this paper, we analyze subject-predicate gender agreement. Gender attraction has been investigated only in a few studies, and mostly in Romance languages, which have two genders. We report one production and three comprehension experiments on Russian, a language with three genders. To the best of our knowledge, this is the first comprehension study looking at agreement attraction in a non-binary category. Below we present several findings from the research on number agreement, which will be most important for our study, and different accounts of attraction. Next, we review the few existing studies on gender attraction, providing rationale for the present work.

#### 1.1.1. Plural markedness effect

In all studied languages, attraction effects were found to be asymmetric. They can be observed when the head is singular, and the attractor is plural [as in (1) above], but are much weaker or virtually non-existent in the opposite configuration. In the majority of agreement attraction studies, this asymmetry is explained in terms of feature markedness. Plural is assumed to be the marked value of number feature^2^, and the asymmetry is attributed to the fact that attractors with a marked feature are more disruptive. Hence it is known under the name of “plural markedness effect.”

However, the concept of markedness is not widely agreed upon. Different authors adopt different theoretical approaches and different tests to determine marked and unmarked feature values [including frequency, presence of a non-zero affix, default use of a form (e.g., in impersonal sentences), various semantic tests etc.; see Haspelmath, 2006]. It is impossible to evaluate them looking only at singular and plural. To figure out which of these properties may be relevant for the asymmetry between feature values (and whether it makes sense to attribute it to markedness in a particular theoretical framework), it is crucial to look at other features systems. As we will show below, Russian gender is interesting in this respect because the results of different markedness tests do not converge, letting us tease several approaches apart.

#### 1.1.2. Parallel results in production and comprehension

Experimental studies demonstrated that attraction exists not only in production, but also in comprehension. In production it manifests itself as agreement errors. In comprehension attraction errors have been observed to trigger more grammaticality judgment mistakes and to provoke less pronounced effects in reading time and EEG studies than other agreement errors. In other words, people perceive ungrammatical sentences as if they were grammatical or had a minor violation. This is often called a “grammaticality illusion.”

The results from production and comprehension are largely parallel (in particular, significant attraction effects are observed only with plural attractors). This is often used to conclude that the mechanism of attraction is the same in both modalities. We will come back to this problem discussing our findings because we did not observe parallelism that we expected based on the previous studies.

#### 1.1.3. Debate on ungrammaticality illusions

We just mentioned that in comprehension, attraction causes grammaticality illusions, making ungrammatical sentences more acceptable. Can it also lead to ungrammaticality illusions, and make grammatical sentences less acceptable? For example, if people tend to miss agreement errors in sentences like (2a), do they sometimes see non-existent errors in sentences like (2b)? As we show below, different approaches to attraction make opposing predictions about ungrammaticality illusions, so this is an important question.

(2)
  - The key to the cabinets were rusty.
  - The key to the cabinets was rusty.

Several studies (e.g., Nicol et al., 1997; Pearlmutter et al., 1999) suggested that ungrammaticality illusions do arise. However, Wagers et al. (2009) demonstrated that at least on-line findings may be artifactual (they might be due to the fact that processing plural nouns carries an additional cost compared to singular ones, not to any aspects of subject-verb agreement processing). This hypothesis can be tested by analyzing some cases where this problem does not apply, and we do so in the present study looking at gender agreement ^3^.

#### 1.1.4. The role of morphophonology

Hartsuiker et al. (2003) showed that when the form of the attractor is morphologically ambiguous and coincides with nominative, the rate of attraction errors increases. They compared German sentences like (3a,3b). People made more errors in (3a), where the attractor (*die Demonstrationen*) is ambiguous between accusative and nominative, compared to (3b), where the attractor (*den Demonstrationen*) is unambiguously dative. We do not explore the role of morphophonology in the present study, but take this factor into account. Several studies also demonstrated that heads with regular inflections are more resistant to attraction, but no similar effects were observed for attractors (e.g., Bock and Eberhard, 1993; Vigliocco et al., 1995).

(3)   a.   die                Stellungnahme gegen  die
              the_*F.NOM.SG*_ position          against the_*F.ACC.PL*_
              Demonstrationen
              demonstrations
        b.   die       Stellungnahme zu den
              the_*F.NOM.SG*_ position       on the_*DAT.PL*_
              Demonstrationen
              demonstrations

### 1.2. Models of agreement attraction

There exist two major approaches to agreement attraction. Here they will be referred to as the “representational account” and the “retrieval account.” Models that belong to the **representational account** share one crucial assumption: agreement attraction takes place because the mental representation of the number feature on the subject NP is faulty or ambiguous (Nicol et al., 1997; Vigliocco and Nicol, 1998; Franck et al., 2002; Eberhard et al., 2005; Staub, 2009, 2010; Brehm and Bock, 2013). In some models, it is assumed that syntactic features can “percolate” or otherwise move to neighboring nodes: for example, sometimes number features from the embedded NP percolate to the subject NP (which normally has the same number marking as its head).

Another model known as Marking and Morphing (Eberhard et al., 2005) postulates that the number value of the subject NP is a continuum, i.e., it can be more or less plural. For example, if a subject NP contains a singular head and a plural dependent NP it is more plural than a subject NP with a singular modifier. A subject NP that is formally singular, but refers to a collective entity is more plural than the ones referring to singular entities. The more plural the subject NP, the higher the possibility of choosing a plural verb. In such accounts there is no way to avoid ungrammaticality illusions: if the agreement controller can be mis-construed or ambiguous, there is no way to restrict such mis-construals to only ungrammatical sentences. They happen even before we encounter the verb, i.e., even before it is clear whether the sentence is or is not grammatical.

Now let us turn to the **retrieval account** (Solomon and Pearlmutter, 2004; Lewis and Vasishth, 2005; Badecker and Kuminiak, 2007; Badecker and Lewis, 2007; Wagers et al., 2009; Dillon et al., 2013). Research on memory suggests that the amount of material a person can hold in a ready-to-process state is extremely limited (McElree, 2006; Cowan, 2001). Thus, it can be hypothesized that when we reach an agreeing predicate, the subject needs to be reactivated. This reactivation can be done via so-called cue-based retrieval (Lewis and Vasishth, 2005; McElree, 2006): we query the memory with a set of cues (e.g.,“number: plural,” “case: nominative” etc.) and select an element that matches the maximum number of cues.

This process is not error-free, and the retrieval account argues that attraction arises at this stage. For example, in a sentence like “The key to the cabinets are rusty” the form of the verb suggests that we need to look for an NP with the features “subject” and “plural.” However, no NP perfectly satisfies these conditions: *key* is the subject, but is not plural, and *cabinets* is plural, but is not the subject. It is hypothesized that in such conditions we may mistakenly select the wrong NP. The retrieval approach predicts the absence of ungrammaticality illusions: if a sentence is grammatical, the true subject is a *perfect* match and will always be selected. Thus, unlike in the representational account, there is nothing wrong or ambiguous in the syntactic structure, errors are access failures. Such cases with several elements competing for retrieval are an instance of “retrieval interference.” Other examples are discussed in Van Dyke and Johns (2012).

### 1.3. Studies of gender agreement attraction

Relatively few studies of gender agreement attraction have been conducted so far. Their results do not always converge, but one thing seems to be certain: attraction effects are present. They have been observed in several experiments on different languages.

#### 1.3.1. Previous studies on languages with two genders

As far as we know, the first attempt to induce gender agreement attraction was made in the production study on Italian by Vigliocco et al. (1995). Virtually no evidence of attraction was found: out of 1920 responses only four (0.2%) contained a gender error. However, in a later study Vigliocco and Franck (1999) observed gender agreement attraction in Italian.

Vigliocco and Franck carried out four production experiments: two on Italian and two on French. Both languages have two genders: masculine and feminine. In all experiments, participants saw a masculine and a feminine adjective at the same time (one above the other) and then a noun phrase, and had to combine them saying the resulting sentence aloud. The gender of the head and the attractor were manipulated. When the genders mismatched, people were found to make more agreement errors. In Italian, there was no significant difference between FM and MF conditions^4^. In French, more errors were made in FM conditions (the difference was significant in Experiment 2 and marginally significant in Experiment 4). Whether the head gender was purely grammatical (on inanimate nouns) or conceptual (on animate nouns) also played a role. Participants made fewer errors in the latter case. Thus, semantic factors do enter the picture in case of gender agreement attraction, but, as far as we can judge, only to suppress it (on the contrary, conceptual numerosity can increase the number agreement attraction rate).

The observed pattern of attraction errors was different from number agreement studies. Firstly, a significant number of errors was made in all mismatch conditions, while in case of number agreement, the error rate in the conditions with plural heads and singular attractors was very low, often the same as the error rate without attraction. Secondly, both in French and in Italian, masculine is used as the grammatical default (for example, it appears in impersonal constructions and in the cases where the predicate must agree with several masculine and feminine nouns) and is more frequent. So the pattern observed in French (more errors in FM conditions) is the reverse of the number agreement attraction pattern found across languages.

The authors concluded that feature markedness does not matter for gender agreement and outlined an explanation based on inflectional differences between Italian and French. However, this explanation was undermined by Anton-Mendez et al. (2002) who conducted a production study on Spanish. Spanish is similar to Italian in terms of adjectival inflections, but the results were the same as in French. In addition to that, Vigliocco and Zilli (1999) and Franck et al. (2008) demonstrated in a number of experiments on Italian, Spanish, and French that the morphophonological properties of the head influence the error rate in gender agreement attraction. As in the studies of number agreement attraction, there were fewer errors when heads had regular inflections, but no similar effects were found for attractors.

We could find only two studies examining gender agreement attraction in comprehension: Acuña-Fariña et al. (2014) and Martin et al. (2014). Both looked at Spanish, eye-tracking was used in the first and ERPs in the second. Attraction effects were detected, but no differences between M and F genders were reported.

#### 1.3.2. Previous studies on languages with three genders

Badecker and Kuminiak (2007) (henceforth, B&K) report results of three production experiments on Slovak. Slovak has three genders: masculine, feminine, and neuter. M is the most frequent, N is the least frequent, but is used in impersonal constructions. In all experiments, participants were given subject NPs (often called “preambles') and asked to generate complete sentences. In Experiment 1, B&K compared the number of errors in two groups of conditions: MM, MF, FF, FM and MM, MN, NN, NM. As in the previous studies, there were significantly more errors in mismatch conditions than in match conditions. But the pattern was different: there were more errors in the MF condition compared to the FM and in the NM compared to the MN.

Experiment 2 confirmed the results of Experiment 1 (it contained MM, MF, FF, and FM conditions and was designed to test the role of morphophonological factors). In Experiment 3, NN, NM, and NF conditions were compared. NM and NF preambles provoked more errors than NN preambles; but the number of errors in NM and NF conditions was comparable. Explaining this pattern, B&K adopt an optimality-theoretic approach and argue that there is no single markedness hierarchy in the Slovak gender system (such as N < M < F), but markedness is defined in pairs (N < M, N < F, M < F). Among other things, the results of this study show that frequency does not play a role for feature asymmetries.

Another production experiment was conducted on Russian (Lorimor et al., 2008). The authors manipulated both the number and the gender of heads and attractors (only M and F genders were used). In all trials, participants saw *and* heard the predicate and then saw the preamble. Their task was to construct a sentence using these two parts and to say it aloud. Out of 1155 answers where gender agreement was necessary (in Russian, as well as in Slovak, verbs agree in gender only in past tense singular forms), only seven (0.6%) contained an agreement error. Based on this, the authors concluded that gender agreement attraction does not exist in Russian.

To summarize, in all gender agreement attraction studies, if any effects are observed, error rates in all mismatch conditions are higher than in match conditions (unlike in number attraction studies, where significant effects are found only in one mismatch condition: with singular heads and plural attractors). Otherwise, the results of gender agreement studies are different: larger effects are found in the FM condition (compared to the MF condition) in Spanish and French, and in the MF and NM conditions (compared to the FM and MN conditions) in Slovak. The results from Slovak are closer to the pattern observed for number, if we assume that feminine and masculine genders and plural number are marked.

Out of several approaches to attraction outlined above, the existence of gender agreement attraction is hardly compatible with the Marking and Morphing model, primarily because in the absolute majority of cases, gender features are semantically empty. Moreover, even if we take nouns with conceptual gender, as *mal'čik* “boy_*M*_” or *sestra* “sister_*F*_” in Russian, it makes little sense to assume that, for example, having an M dependent NP could make an F noun “more masculine.” Notably, we do not want to say that the existence of attraction with semantically empty features implies that conceptual numerosity cannot play any role for number agreement attraction - various experimental findings clearly indicate that it does (e.g., Bock and Cutting, 1992; Eberhard, 1999; Haskell and MacDonald, 2005; Mirkovic and MacDonald, 2013). We would only like to stress that attraction is possible without any semantic effects of this sort and therefore should result from some process that does not depend on them (e.g., from the formal properties of features). Semantic effects can be added to the picture, but this is optional.

### 1.4. The present study

Apparently, gender agreement attraction errors are more difficult to induce than number errors. For example, Vigliocco et al. (1995) did not observe them in Italian, although they were found in subsequent experiments. So we decided to run another production experiment on Russian replicating B&K's first experiment on Slovak (which, in terms of its gender system, is very close to Russian). Our goal was to see whether any attraction errors would be induced, and, if yes, whether the pattern would be similar to B&K's study or to what has been observed for French, Spanish, or Italian. We also planned comprehension experiments because no existing studies had looked at comprehension in a language with three genders. We were particularly interested to find out whether production and comprehension results would be parallel and whether ungrammaticality illusions would be found. Before we move on to the experiments, let us present a brief overview of the Russian gender system.

#### 1.4.1. Russian gender system

Russian nouns are inflected for number and case, and the ones that have the same endings in the majority of forms are grouped into declension classes. Russian has three declension classes for nouns (and a separate class for substantivized adjectives). The first class includes almost all M nouns (they have zero endings in nominative singular, like *mal'čik* “boy”) and all N nouns (they have -*o* or -*e* endings, like *okno* “window”). These M and N nouns use the same set of endings in all cases except for genitive plural and nominative and accusative in singular and plural (in plural, all declension classes have the same endings in dative, instrumental and locative). The second class includes the majority of F nouns (they end in -*a* or -*ja*, like *devočka* “girl”) and a small group of animate M nouns with the same endings, like *mužčina* “man.” The third class includes F nouns with zero endings in nominative singular, like *doč'* “daughter.” In addition to that, there are some irregular and uninflected nouns.

Thus, in most cases, it is impossible to determine the gender of the noun unambiguously looking at the noun itself, and, at least prima facie, we cannot speak of something like morphological markedness in the noun system. Let us add that M nouns are the most frequent and N nouns are the least frequent. M nouns constitute about a half of the lexicon, F nouns - about 30–35%, N nouns are the rest (Yanovich and Fedorova, 2006; Slioussar and Samoilova, 2014).

Gender agreement can be observed only in singular, on adjectives, participles and past tense verb forms. Russian adjectives and participles have so-called full forms (used attributively and predicatively) and short forms (used only in predicates and inflected for number and gender, but not for case). M form is the citation form (i.e., the form would appear in dictionaries, grammatical descriptions etc.).

Verb forms and short forms of adjectives and participles have zero endings in M gender (e.g., *byl* “was_*M*_” - *byla* “was_*F*_” - *bylo* “was_*N*_”), otherwise all forms have non-zero endings (e.g., *krasivyj* “beautiful_*M*.*NOM*.*SG*_” - *krasivaja* “beautiful_*F*.*NOM*.*SG*_” - *krasivoe* “beautiful_*N*.*NOM*.*SG*_”). Thus, we cannot say that M forms are morphologically unmarked, even if we limit ourselves to predicates. In impersonal sentences, where unmarked forms are expected, N predicates are used, as (4) shows.

(4)     Svetalo.
          dawn_*PST.N.SG*_
          It dawned.

As for gender conflict resolution, another classical test for markedness, it is of limited use in Russian because there is no gender agreement in plural. Gender conflict resolution can be observed only in constructions like “X and Y each did something.” We conducted an informal questionnaire, asking about 30 native speakers.

As we discuss below, acceptability of such sentences differs depending on animacy of the nouns and the genders that are combined, and there is substantial individual variation among speakers. However, one crucial generalization can be made: examples with the feminine or neuter forms of *každyj* “each' are never found even marginally acceptable, only some examples with the masculine forms are.

Firstly, let us consider sentences with M and F nouns, like in (5). Not all speakers of Russian find these examples acceptable, but for those who do, this construction sounds better with human animates (5a) than with non-human animates (5b). Nobody accepts this construction with inanimate nouns, as in 6a), although they can be used in such sentences if both nouns are of the same gender, as in (6b)^5^.

(5)   a.   Mužčina        i       ženščina    každyj
              man_*M*.*NOM*.*SG*_ and woman_*F*.*NOM*.*SG*_ each_*M*.*NOM*.*SG*_
              sjeli         po             jabloku
              ate_*PST*.*PL*_ PREP_*DISTR*_ apple_*DAT*.*SG*_
        b.   Jož                     i         svin'ja
              hedgehog_*M*.*NOM*.*SG*_ and swine_*F*.*NOM*.*SG*_
              každyj    sjeli                    po         jabloku.
              each_*M*.*NOM*.*SG*_ ate_*PST*.*PL*_ PREP_*DISTR*_ apple_*DAT*.*SG*_

(6)   a.   Divan              i          krovat'    každyj
              sofa_*M*.*NOM*.*SG*_ and bed_*F*.*NOM*.*SG*_ each_*M*.*NOM*.*SG*_
              stoili    celoe    sostojanie.
              cost_*PST*.*PL*_ whole_*ACC*.*SG*_ fortune_*ACC*.*SG*_
        b.   Kušetka           i          krovat'    každaja
              couch_*F*.*NOM*.*SG*_ and bed_*F*.*NOM*.*SG*_ each_*F*.*NOM*.*SG*_
              stoili    celoe    sostojanie.
              cost_*PST*.*PL*_ whole_*ACC*.*SG*_ fortune_*ACC*.*SG*_

Now let us look at M and N nouns. More than half of the speakers we asked rejected this construction even with animate human nouns (7a) as ungrammatical, but those who accepted it used masculine form. All our informants rejected examples with non-human animates like (7b) or found them only marginally acceptable. This might be at least partly due to independent factors (the relevant neuter words, like *mlekopitajuščee* “mammal,” ž*ivotnoe* “animal,” *nasekomoe* “insect,” tend to be abstract), but is still telling.

(7)   a.   Voin                      i          dit'a    každyj
               warrior_*M*.*NOM*.*SG*_ and child_*N*.*NOM*.*SG*_ each_*M*.*NOM*.*SG*_
               sjeli           po         jabloku.
               ate_*PST*.*PL*_ PREP_*DISTR*_ apple_*DAT*.*SG*_
        b.   Gryzun               i          nasekomoe    každyj
               rodent_*M*.*NOM*.*SG*_ and insect_*N*.*NOM*.*SG*_ each_*M*.*NOM*.*SG*_
               vypili            po         kaple.
               drank_*PST*.*PL*_ PREP_*DISTR*_ drop_*DAT*.*SG*_

Finally, such constructions with F and N nouns, as in (8), were rejected by most of our informants. The few people who accepted them again preferred the masculine form.

(8)     Ženščina             i       dit'a    každyj
          woman_*F*.*NOM*.*SG*_ and child_*N*.*NOM*.*SG*_ each_*M*.*NOM*.*SG*_
          sjeli          po         jabloku.
          ate_*PST*.*PL*_ PREP_*DISTR*_ apple_*DAT*.*SG*_

Let us add that M nouns are used to refer to groups of people of mixed or uncertain gender, or to an arbitrary member of such groups. This generalization is discussed by Yanovich (2012) who shows that it does not hold for animals. For example, the word *sobaka* “dog” is feminine. There are specific words to denote male and female dogs, but they are much more often used as swearwords, like the English *bitch*. To sum up, N appears to be the grammatical default as the gender used in impersonal constructions, while all cases where M is used as the standard option are limited to the nouns denoting humans and sometimes other animates. In all our experiments, we used only inanimate nouns as heads and attractors (we wanted to avoid additional factors before the general picture becomes clear)^6^.

## 2. Experiment 1

Experiment 1 was designed to check whether the findings of Badecker and Kuminiak (2007) would be replicated in Russian, which is very close to Slovak in the relevant part of the grammar. In particular, both languages have three genders, M is the most frequent, N is the least frequent, but is used in impersonal sentences. There are no articles. Gender agreement can be observed on adjectives and participles (in singular) and on verbs (in past tense singular). The system of declensions is very similar as well.

### 2.1. Participants

Thirty native speakers of Russian (8 male, 22 female) participated in Experiment 1. Ages ranged from 18 to 50 (mean age 28.7, *SD* 9.4). No participant took part in more than one experiment. All experiments reported in this paper were carried out in accordance with the Declaration of Helsinki and the existing Russian and international regulations concerning ethics in research. All participants provided informed consent. They were tested at the Laboratory for Cognitive Studies of Saint-Petersburg State University.

### 2.2. Materials

In this experiment, participants first saw a predicate, then on the next slide a subject at which point they were asked to produce a complete sentence. In half of the cases, predicates did not agree with the subject in gender, and participants were asked to modify them. Like in B&K's study, subject noun phrases were always built according to the following schema: NP_1_–preposition–NP_2_, e.g., *okno vo dvor* “window_*N*.*SG*_ to yard_*M*.*SG*_.” NP_1_ was always in nominative singular, NP_2_ was in accusative singular. We selected inanimate nouns that have the same form in accusative and nominative, since this was shown to inflate the error rate (Badecker and Kuminiak, 2007). As in many other agreement attraction studies, we had both adjunct and argument PPs.

The predicates always consisted of two words: the copula *byt'* “to be” in the past tense (where gender agreement can be observed) and an adjective or participle. We opted for such predicates because they are short and do not contain any objects or other nouns that could cause additional disturbance of subject-predicate agreement (initially, we wanted to use single verbs, but could not come up with such predicates for all experimental stimuli). Adjectives and participles were always in instrumental singular form^7^.

The genders of NP_1_ and NP_2_ were manipulated. As Table 1 shows, these two factors were not fully crossed. Like in B&K's Experiment 1, we used only seven out of nine possible combinations of genders. Additionally, we manipulated the agreement marking on the predicate^8^. Sample stimuli in conditions 1-4 in Table 1 represent one set: two variants of the subject NP (one head and two different dependent nouns, or attractors) and two variants of the predicate (matched or mismatched in gender with the subject). We constructed 48 sets, 12 for each of the four combinations of conditions. This approach to the construction of materials (one head noun and several attractors of different genders, plus a grammatical and an ungrammatical version of the predicate) holds for all experiments in this article. All materials are listed in Appendices in Supplementary Material.

**Table 1:** **Gender combinations used in Experiment 1**.

**Condition**	**NP_1_ gender**	**NP_2_ gender**	**Predicate gender**	**Example**
1 / 2	M	M	M / F	*byl prosročennym / byla prosročennoj + recept na porošok*
				(was_*M*.*SG*_ expired_*M*.*SG*_ / was_*F*.*SG*_ expired_*F*.*SG*_ + prescription_*M*.*NOM*.*SG*_ for powder_*M*.*ACC*.*SG*_)
3 / 4	M	F	M / F	*byl prosročennym / byla prosročennoj + recept na maz'*
				(was_*M*.*SG*_ expired_*M*.*SG*_ / was_*F*.*SG*_ expired_*F*.*SG*_ + prescription_*M*.*NOM*.*SG*_ for ointment_*F*.*ACC*.*SG*_)
5 / 6	F	F	F / M	
7 / 8	F	M	F / M	
9 / 10	M	M	M / N	
11 / 12	M	N	M / N	
13 / 14	N	N	N / M	*byl otkrytym / bylo otkrytym + okno v pole*
				(was_*M*.*SG*_ opened_*M*.*SG*_ / was_*N*.*SG*_ opened_*N*.*SG*_ + window_*N*.*NOM*.*SG*_ to field_*N*.*ACC*.*SG*_)
15 / 16	N	M	N / M	*byl otkrytym / bylo otkrytym + okno vo dvor*
				(was_*M*.*SG*_ opened_*M*.*SG*_ / was_*N*.*SG*_ opened_*N*.*SG*_ + window_*N*.*NOM*.*SG*_ to yard_*M*.*ACC*.*SG*_)

In addition to that, we constructed 100 fillers, also consisting of a predicate and a subject. Subject NPs had singular or plural heads and adjectival or prepositional modifiers (the NPs inside these PPs were not in accusative). Predicates were similar to the ones in target stimuli and did not agree with subjects in gender in one third of the cases.

Each participant saw only one target stimulus from each set. Consequently, we had four experimental lists with 148 items (48 stimuli and 100 fillers). The number of conditions was balanced for every list. Thus, every participant saw three target items per condition: for example, three FF stimuli (having an F head and an F attractor) with a matched F predicate, three FF stimuli with a mismatched M predicate etc. All lists began with ten fillers, and then fillers and experimental items were presented in a pseudo-random order, with the constraint that no more than two experimental items occur consecutively.

### 2.3. Procedure

In a pilot experiment, we used the same procedure as in B&K 's study: participants listened to preambles and were asked to generate complete sentences. But after running six subjects, we did not get any attraction errors. This can be explained by the fact that such errors are in general relatively infrequent. In B&K 's study, they occurred in 3% cases on average. Since the number of errors varies from subject to subject, the probability to elicit no errors from several people in a row is considerably high. However, we decided to switch to a different method in the main experiment in hope to elicit more errors.

The experiment was run on a Macintosh computer using PsyScope software (Cohen et al., 1993). In every trial, participants saw on the computer screen a fixation point (for 300 ms), then a predicate (for 800 ms), and then a subject NP (for 800 ms). Their task was to combine the predicate and the subject in a grammatical sentence and to say it aloud. If the predicate did not agree with the subject, participants were instructed to modify the predicate. Before the main session started, the experimenter explained the task on two sample items (saying that participants would see two phrases and would be asked to combine them into a correct sentence as fast as possible, i.e., without explicitly mentioning gender agreement). Then there were four practice items.

To encourage participants to respond faster, a time counter appeared on the screen after both the predicate and the subject were presented. As soon as the participant responded, the experimenter pressed a key, and the next trial started. All participants' responses were tape-recorded. An experimental session lasted around 7.5 min.

### 2.4. Results

The participants' responses were transcribed, and each of them was assigned into one of the following categories:

- Correct response: the sentence is grammatical, the subject and the predicate provided as stimuli are repeated faithfully.
- Agreement error: the sentence is correct except for a gender agreement error.
- Repetition error: the sentence is grammatical, but the subject or the predicate is repeated incorrectly (for example, the word *krem* “cream” was used instead of the word *maz'* “ointment”).
- A combination of a repetition error and an agreement error.
- Incomplete response: the participant utters only a part of the sentence or says nothing at all.
- A combination of an incomplete response and an agreement error: the sentence is incomplete, but a verb, a participle or an adjective was uttered and did not agree with the subject (cf. 9a – 9b).
  (9)   a.   Recept                 na    maz'                byla    … …
                 recipe_*M*.*NOM*.*SG*_ for ointment_*F*.*ACC*.*SG*_ was_*F*.*SG*_
          b.   Recept                 na       maz'
                 recipe_*M*.*NOM*.*SG*_ for ointment_*F*.*ACC*.*SG*_
                 prosročennaja…
                 expired_*F*.*SG*_               …

Errors in subject-verb gender agreement were the only grammar errors participants made, all other errors involved incorrectly repeating or omitting lexical material (we did not expect any other grammar errors, for example, in number or case, but they could have occurred accidentally). To exclude mishearings during transcription, both authors of this paper and two other native speakers of Russian listened to all responses to target stimuli. The number of errors in each category is given in Table 2. In case of self-corrections, only the first variant was counted, both when participants changed an answer with an error to a correct one and when they did the opposite (this happened in three cases).

**Table 2:** **The distribution of responses in Experiment 1**.

Correct responses	1018 (71.2%)
Agreement errors	61 (4.3%), 8 of them partial^a^
Repetition errors	111 (7.8%)
Repetition and agreement errors	9 (0.6%), 3 of them partial
Incomplete responses	224 (15.7%)
Incomplete responses with agreement errors	7 (0.5%)

a *In most sentences with agreement errors, both the verb and the adjective or participle were in a wrong form. They are components of a complex predicate, so we counted this as one error (note that counting them as two errors instead would not affect the outcome, because the differences between the relevant conditions would only be inflated). However, in several cases only one of the two components did not agree with the subject*.

At the following stage of analysis, we collapsed all agreement errors together. The distribution of errors by experimental conditions is given in Table 3. In total, there were 77 agreement errors (5.4% from all responses). Only 13 out of them were not due to attraction (they are discussed in more detail below). The difference between the number of agreement errors with and without attraction is statistically significant according to the chi-square test^9^ [$\chi_{\left(1,\;N\;=\;77\right)}^{2}=18.97$, *p* <0.01], so our results show that gender agreement in Russian is subject to attraction.

**Table 3:** **The distribution of responses by condition in Experiment 1**.

	**Correct response**	**Agr. error (attraction)**	**Agr. error (no attraction)**	**Other errors**
Condition 1 (MM + M)^a^	69	0	0	16
Condition 2 (MM + F)	69	0	0	16
Condition 3 (MF + M)	66	3	1	20
Condition 4 (MF + F)	53	19	1	17
Condition 5 (FF + F)	65	0	0	25
Condition 6 (FF + M)	57	0	3	30
Condition 7 (FM + F)	66	1	0	23
Condition 8 (FM + M)	50	10	1	29
Condition 9 (MM + M)	74	0	0	16
Condition 10 (MM + N)	64	0	3	23
Condition 11 (MN + M)	69	1	0	20
Condition 12 (MN + N)	59	11	0	20
Condition 13 (NN + N)	64	0	1	25
Condition 14 (NN + M)	68	0	2	20
Condition 15 (NM + N)	62	6	1	21
Condition 16 (NM + M)	63	13	0	14

a *Due to our mistake, there are 85 responses in conditions 1 and 2 rather than 90*.

As Table 3 shows, agreement errors were more frequent in predicate mismatch conditions, but were not limited to them. Out of 13 errors without attraction, in eight cases, a mismatched predicate was not changed, but there were also five cases where participants produced a neuter predicate with an MF subject, a masculine predicate with an NN subject etc., although they were provided with other forms, matched or mismatched with the subject. Out of 64 attraction errors, 11 errors occurred in predicate match conditions, i.e., participants changed the correct gender of the predicate they were provided with to an incorrect one due to attraction.

Conditions with matched and mismatched predicates are collapsed in Table 4 showing that the number of agreement attraction errors differs depending on the combination of genders of the head and attractor nouns. To test whether these differences are statistically significant, we modeled the data with a mixed-effects logistic regression in the statistical software program R (R Core Team, 2014) using the *glmer* function from the *lme4* package (Bates et al., 2015).

**Table 4:** **The Number of gender agreement attraction errors by condition in Experiment 1**.

**Head/attractor gender**	**Correct responses**	**Attraction errors**	**Other errors**
MF	119	22	39
FM	116	11	53
MN	136	12	42
NM	125	19	36

Firstly, we compared MF and FM conditions. The logistic regression evaluated the likelihood of an agreement attraction error (coded as 1) vs. a correct response (coded as 0). The combination of genders was treated as a fixed effect. For the predictors we used contrast coding: MF was coded as 0.5, FM was coded as −0.5. Random intercepts by participant and by item were also included in the model. The results of the analysis are reported in Table 5. The coefficient for the intercept was significant, reflecting that most responses were correct. There was also a significant main effect of Gender Combination indicating that F attractors trigger significantly more errors than M attractors.

**Table 5:** **Results of the analysis for Experiment 1**.

**Conditions**	**Predictor**	**Coefficient**	**Std. error**	**Wald Z**	***p***
MF vs. FM	(Intercept)	−3.04	0.43	−7.01	<0.01
	GenComb	−0.95	0.48	−1.96	0.05
MN vs. NM	(Intercept)	−2.68	0.30	−8.82	<0.01
	GenComb	0.62	0.39	1.59	0.11

Secondly, we compared MN and NM conditions in the same way. MN was coded as 0.5, NM was coded as −0.5. The coefficient for the intercept was again significant because most responses were correct. But the main effect of gender combination did not reach significance. We also compared MF and MN conditions and FM and NM conditions, as well as the number of non-agreement (“other”) errors in different conditions, but did not find any significant differences.

### 2.5. Discussion

The results of Experiment 1 are similar to the results of B&K's first experiment, which can be explained by the fact that the two languages have similar gender systems, as we demonstrated in the introduction. In both studies, F attractors triggered more errors than M attractors. N attractors triggered fewer errors than M attractors, but this difference was statistically significant only in B&K's study. As we mentioned in the introduction, other authors studying gender attraction in French and Spanish (which have two genders and where M is grammatical default), observed a different pattern: there were more errors with M attractors than with F attractors. We postpone further discussion until the general discussion section.

## 3. Experiment 2a

Experiment 2a was designed to find out whether gender agreement attraction can also be detected in comprehension. For the sake of comparison with Experiment 1, we used the same combinations of head and attractor noun genders.

### 3.1. Participants

Forty-eight native Russian speakers (19 female and 29 male) took part in the experiment. Ages ranged from 19 to 26 (mean age 20.9, *SD* 1.9).

### 3.2. Materials

The materials consisted of target and filler sentences. All target sentences were 9–10 words long and followed the schema: NP_1_–preposition–NP_2_–copula (*byt'*) - adjective/participle - four-five words modifying the predicate. We had the same 16 conditions as in Experiment 1 (see Table 1 above). Almost all subject NPs and predicates were based on the materials from Experiment 1 and followed the same constraints. In half of the conditions, the predicate did not agree with the subject. Given existing findings on number agreement attraction, we expected parallel results in production and comprehension. In particular, we expected to find grammaticality illusions in conditions MFF, FMM, MNN, and NMM (this would mean that they would be read significantly faster than the other four ungrammatical conditions: MMF, FFM, MMN, NNM).

As in Experiment 1, conditions were grouped in sets, each set containing four conditions with the same head nouns. An example of a stimuli set is given in (10)^10^. For each condition set we constructed 12 sentences, 48 target sentences in total.

(10)   a.  Recept                 na      porošok              byl
               recipe_*M*.*NOM*.*SG*_ for powder_*M*.*ACC*.*SG*_ was_*M*.*SG*_
               pom'atym    iz-za    sil'nogo
               crumpled_*M*.*SG*_ due.to strong_*GEN*.*SG*_
               volnenija                 pacienta.
               nervousness_*GEN*.*SG*_ patient_*GEN*.*SG*_
              “The recipe for the powder was crumpled due to the
               patient's extreme nervousness.”
         b.   Recept                 na    maz'              byl
               recipe_*M*.*NOM*.*SG*_ for ointment_*F*.*ACC*.*SG*_ was_*M*.*SG*_
               pom'atym    iz-za    sil'nogo
               crumpled_*M*.*SG*_ due.to strong_*GEN*.*SG*_
               volnenija                 pacienta.
               nervousness_*GEN*.*SG*_ patient_*GEN*.*SG*_
         c.   Recept                 na    porošok              byla
               recipe_*M*.*NOM*.*SG*_ for powder_*M*.*ACC*.*SG*_ was_*F*.*SG*_
               pom'atoj    iz-za    sil'nogo
               crumpled_*F*.*SG*_ due.to strong_*GEN*.*SG*_
               volnenija                 pacienta.
               nervousness_*GEN*.*SG*_ patient_*GEN*.*SG*_
         d.   Recept                 na    maz'              byla
               recipe_*M*.*NOM*.*SG*_ for ointment_*F*.*ACC*.*SG*_ was_*F*.*SG*_
               pom'atoj    iz-za    sil'nogo
               crumpled_*F*.*SG*_ due.to strong_*GEN*.*SG*_
               volnenija                 pacienta.
               nervousnessx_*GEN*.*SG*_ patient_*GEN*.*SG*_

Additionally, we constructed 120 fillers, which had roughly the same structure as experimental sentences. Subject NPs in fillers consisted of a single noun modified by an adjective, or of a complex NP, where the embedded noun was not in accusative. All fillers were grammatical. Thus, we had 24 ungrammatical and 144 grammatical sentences, making the grammatical-to-ungrammatical ratio 6:1. Experimental sentences and fillers were distributed in four counterbalanced experimental lists. Every list started with ten fillers; then stimuli and fillers were presented in pseudo-random order with the constraint that a maximum of two stimuli could occur consecutively.

### 3.3. Procedure

The sentences were presented on a PC using Presentation software (http://www.neurobs.com). We used the word-by-word self-paced reading methodology (Just et al., 1982). Each trial began with a sentence in which all words were masked with dashes while spaces and punctuation marks remained intact. Participants were pressing the space bar to reveal a word and re-mask the previous one. One third of the sentences was followed by forced choice comprehension questions to ensure that the participants were reading properly. Two answer variants were presented on the left and on the right of the screen. Participants pressed “f” to choose the answer on the left, and “j” to choose the answer on the right. Participants were instructed to read at a natural pace and answer questions as accurately as possible. They were not informed in advance that sentences would contain errors. An experimental session lasted around 14 min.

### 3.4. Results

We analyzed participants' question-answering accuracy and reading times. Two participants answered more than 20% questions incorrectly, so their data were discarded. Otherwise no participant made more than two mistakes when answering questions to target sentences (i.e., 10% at most). Reading times that exceeded a threshold of 2.5 standard deviations, by region and condition, were excluded (Ratcliff, 1993). For two participants, this led to the exclusion of more than 15% responses, so we did not include their data in further analysis.

**Figure 1:**
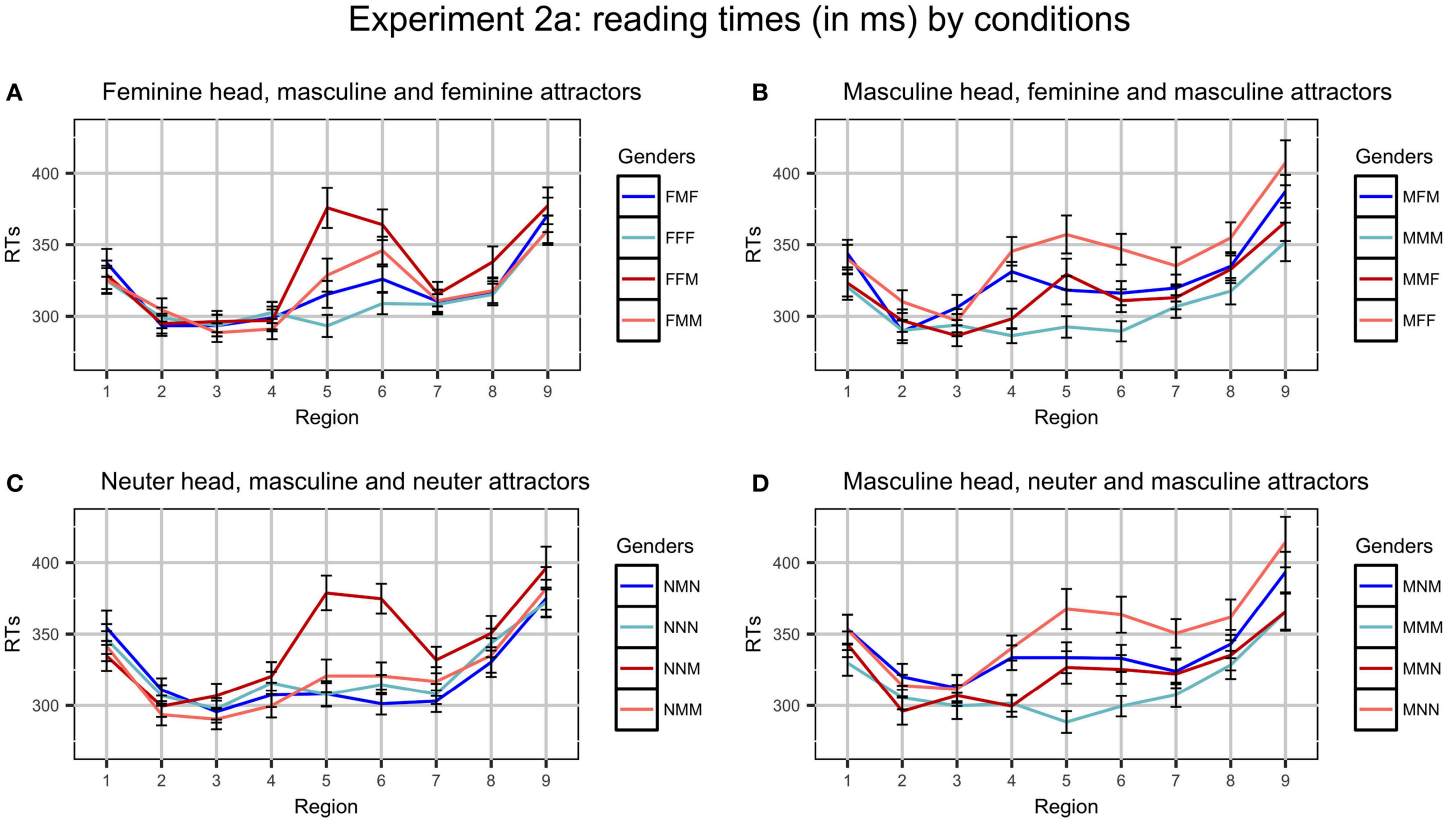
**Plots of mean RTs (in ms) by conditions in Experiment 2a**. Error bars represent standard errors of the means. Regions: NP_1_ (1) - preposition (2) - NP_2_ (3) - copula *byt'* (4) - Adj/Part (5) - spillover (6–9). Ungrammatical conditions are red, grammatical ones are blue. Conditions where the gender of the attractor and the predicate coincide (for example, FFF and FMM) have dark colors, conditions where they do not (for example, FMF and FFM) have light colors. **(A)** Feminine head, masculine and feminine attractors, **(B)** Masculine head, feminine and masculine attractors, **(C)** Neuter head, masculine and neuter attractors, and **(D)** Masculine head, neuter and masculine attractors.

After four participants were excluded, we had 44 participants (11 in each experimental list). In total, 2.3% of the data were excluded as outliers (never more than 3.6% per region and condition). Average RTs per region in different conditions are presented in Figure 1.

The data for each set of conditions (e.g., MMM - MFM - MMF - MFF) were entered in a 2 × 2 Repeated Measures ANOVA with grammaticality and gender match between the attractor and the head nouns as factors. We used IBM SPSS software (www.ibm.com/software/analytics/spss/). Analyses by items and by participants were performed. Data from all regions were tested, but there were significant results only in regions 4–6 in the conditions with M heads and in regions 5–6 in the conditions with F and N heads. Region 4 is the copula, region 5 is an adjective or participle, regions 6–10 contain several words modifying the predicate. The results of the tests for the relevant regions are given in Table 6.

**Table 6:** **Results of the analysis for Experiment 2a**.

**Conditions**	**Region**	**Factor**	**df**	**MS_*effect*_**	**F_1_**	***p***	**df**	**MS_*effect*_**	**F_2_**	***p***
FF vs. FM	5	Gram	**1.43**	**103775.64**	**18.29**	<**0.01**	**1.11**	**35056.83**	**17.86**	<**0.01**
		GenMatch	1.43	5355.95	1.56	0.22	1.11	3326.67	2.05	0.18
		Gram ^*^ GenMatch	**1.43**	**50162.64**	**20.54**	<**0.01**	**1.11**	**21717.52**	**5.01**	**0.05**
	6	Gram	**1.43**	**62551.84**	**21.23**	<**0.01**	**1.11**	**16335.63**	**18.42**	<**0.01**
		GenMatch	1.43	0.05	<0.01	1.00	1.11	0.05	<0.01	1.00
		Gram ^*^ GenMatch	**1.43**	**14823.13**	**4.65**	**0.04**	1.11	3398.65	2.76	0.13
NN vs. NM	5	Gram	**1.43**	**78213.55**	**28.70**	<**0.01**	**1.11**	**20398.13**	**8.87**	**0.01**
		GenMatch	**1.43**	**33363.06**	**10.93**	<**0.01**	1.11	9996.53	1.92	0.19
		Gram ^*^ GenMatch	**1.43**	**36720.35**	**16.67**	<**0.01**	**1.11**	**11405.25**	**29.57**	<**0.01**
	6	Gram	**1.43**	**71017.35**	**32.99**	<**0.01**	**1.11**	**18794.17**	**27.45**	<**0.01**
		GenMatch	**1.43**	**51758.70**	**26.98**	<**0.01**	1.11	12558.27	1.99	0.19
		Gram ^*^ GenMatch	**1.43**	**20026.31**	**14.92**	<**0.01**	**1.11**	**4945.08**	**7.40**	**0.02**
MM vs. MF	4	Gram	**1.43**	**8423.58**	**5.21**	**0.03**	1.11	2100.13	2.12	0.17
		GenMatch	**1.43**	**93656.82**	**58.58**	<**0.01**	**1.11**	**25002.51**	**8.46**	**0.01**
		Gram ^*^ GenMatch	1.43	70.01	0.05	0.83	1.11	0.05	<0.01	0.99
	5	Gram	**1.43**	**63205.83**	**16.87**	<**0.01**	**1.11**	**19008.48**	**16.83**	<**0.01**
		GenMatch	**1.43**	**34672.63**	**10.56**	<**0.01**	1.11	9163.21	1.11	0.32
		Gram ^*^ GenMatch	1.43	114.41	0.02	0.88	1.11	19.25	0.02	0.89
	6	Gram	**1.43**	**32730.00**	**13.37**	<**0.01**	**1.11**	**8554.68**	**9.06**	**0.01**
		GenMatch	**1.43**	**47491.25**	**27.90**	<**0.01**	1.11	12185.81	2.24	0.16
		Gram ^*^ GenMatch	1.43	2128.79	0.88	0.35	1.11	401.36	1.17	0.30
MM vs. MN	4	Gram	1.43	1264.21	0.75	0.39	1.11	231.00	0.38	0.55
		GenMatch	**1.43**	**66406.31**	**24.34**	<**0.01**	1.11	15699.95	2.97	0.11
		Gram ^*^ GenMatch	1.43	2116.29	1.88	0.18	1.11	321.89	1.34	0.27
	5	Gram	**1.43**	**63247.53**	**20.26**	<**0.01**	**1.11**	**16965.12**	**23.11**	<**0.01**
		GenMatch	**1.43**	**86314.12**	**18.61**	<**0.01**	1.11	22733.11	2.23	0.16
		Gram ^*^ GenMatch	1.43	414.21	0.13	0.72	1.11	1.02	< 0.01	0.96
	6	Gram	**1.43**	**36279.68**	**9.04**	<**0.01**	**1.11**	**9509.07**	**7.94**	**0.02**
		GenMatch	**1.43**	**52540.19**	**14.24**	<**0.01**	1.11	15123.00	1.76	0.21
		Gram ^*^ GenMatch	1.43	29.05	0.01	0.93	1.11	40.33	0.02	0.90

*Analyses with p ≤ 0.05 are shown in bold*.

#### 3.4.1. Feminine head, masculine attractor

The main effect of Grammaticality is significant in analysis by subjects and by items in regions 5–6, reflecting the fact that ungrammatical sentences were read slower than grammatical ones. The main effect of Gender Match is not significant in any region. The interaction of Grammaticality and Gender Match is significant in analysis by subjects and by items in region 5 and only in analysis by subjects in region 6. Ungrammatical sentences were read faster if the head and the attractor were mismatched in gender (i.e., in the FMM condition compared to the FFM condition). This is the classical attraction pattern.

#### 3.4.2. Neuter head, masculine attractor

The main effect of Grammaticality is significant in regions 5–6, reflecting longer RTs in ungrammatical conditions. The main effect of Gender Match is significant only in analysis by subjects in regions 5–6. The interaction of Grammaticality and Gender Match is significant in regions 5–6, which is again a reflection of the classical attraction pattern: NMM condition was read faster than NNM and, in fact, almost as fast as grammatical conditions.

#### 3.4.3. Masculine head, feminine attractor

The main effect of Grammaticality is significant in analysis by subjects in region 4 and in analysis by subjects and by items in regions 5–6. This reflects the fact that RTs were longer in ungrammatical conditions. The main effect of Gender Match is significant in analysis by subjects and by items in region 4, and only in analysis by subjects in regions 5–6. This corresponds to longer RTs in conditions where the genders on the nouns were mismatched. The interaction of Grammaticality and Gender Match did not reach significance in any regions, which points to the absence of agreement attraction.

#### 3.4.4. Masculine head, neuter attractor

The main effect of Grammaticality is significant in analysis by subject and by items in regions 5–6: the ungrammatical conditions are read slower than grammatical. The main effect of Gender Match is significant only in analysis by subjects in regions 4–6. The interaction of Grammaticality and Gender Match is not significant in any region, so these conditions also show no agreement attraction.

### 3.5. Discussion

As can be seen from the analyses, the results fall into two groups. In the conditions with F or N heads and M attractors there is clear evidence for gender agreement attraction. RTs exhibit the classical attraction profile with grammaticality illusions: ungrammatical sentences where the attractor and the predicate have the same gender (FMM and NMM) are read faster than other ungrammatical sentences (FFM and NNM). Discussing comprehension studies of number agreement attraction in the introduction, we outlined different approaches to this phenomenon, but will opt for one of them ourselves only in the general discussion section once all experimental findings are presented. Let us also note that ungrammaticality illusions are absent: in the sentences with N heads there are virtually no differences between grammatical conditions; in the sentences with F heads, they are insignificantly small.

On the other hand, the conditions with M heads and F or N attractors do not show any evidence of attraction. Both grammatical and ungrammatical sentences where the head and the attractor match in features (MMM, MMF, and MMN) are read faster than the sentences where they are mismatched (MFM, MNM, MFF, and MNN). In case of ungrammatical sentences, this pattern is *the reverse* of what we usually see in attraction cases.

Looking for an explanation of such pattern, we discovered that we need to rule out an important confound first. Unfortunately, we made a mistake during the preparation of experimental materials, and the frequencies of attractors in conditions with M heads were not well balanced. Since this could influence the results in some unexpected way, we conducted an additional experiment where the frequencies were carefully controlled. Conditions with F and N heads did not have this problem, and the results reported for them hold.

## 4. Experiment 2b

In this experiment we follow up on potential frequency effects in the conditions with M heads from Experiment 2a.

### 4.1. Participants

Thirty-five native Russian speakers (17 female, 18 male) took part in the experiment. Ages ranged from 21 to 47 (mean age 31.3, *SD* 6.2).

### 4.2. Materials

We constructed 32 sets of stimuli according to the same schema as in Experiment 2a and observing the same constraints. Head nouns were always masculine. In 16 sets, the attractors were masculine and neuter; in the other 16 sets, the attractors were masculine and feminine. Most of the head nouns were re-used from the Experiment 2a, but we replaced attractors so that their frequencies were closely matched inside the two groups of conditions. We used *The Frequency Dictionary of Modern Russian Language* (Lyashevskaya and Sharoff, 2009). Average frequencies of head and attractor nouns in Experiments 2a and 2b are shown in Table 7. As in Experiment 2a, half of the predicates did not agree with the subject in gender. Additionally, we used 80 fillers from Experiment 2a. Experimental sentences were distributed into four experimental lists, with factors counterbalanced. As a result, we had 112 sentences per list (16 ungrammatical and 96 grammatical), making the grammatical-to-ungrammatical ratio 6:1.

**Table 7:** **Frequencies of the attractors used in Experiments 2a and 2b (in ipm, or instances per million)**.

**Experiment**	**Head gender**	**Attractor gender**	**Mean attractor freq (ipm)**
Experiment 2a	F	F	138.1
		M	120.0
	N	N	105.9
		M	81.7
	M	M	91.8
		F	41.1
	M	M	134.9^a^
		N	78.9
Experiment 2b	M	M	61.4
		F	61.9
	M	M	69.2
		N	68.1

a *It should be noted that one really frequent M noun influences this number a lot. If we get rid of it and of the corresponding N attractor, the frequencies become very close: 73.9 for M attractors and 84.6 for N attractors*.

### 4.3. Procedure

The procedure was the same as in Experiment 2a. An experimental session lasted around 9 min.

### 4.4. Results

Like in Experiment 2a, we analyzed participants' question-answering accuracy and reading times. At the first stages of analysis, the data from three participants were discarded: one of them had <75% accuracy in comprehension questions; the other two read too slowly compared with the others, so more than 15% of their RTs would have to be excluded as outliers (exceeding the threshold of 2.5 standard deviations). As a result, we had 32 participants, eight for each experimental list.

**Figure 2:**
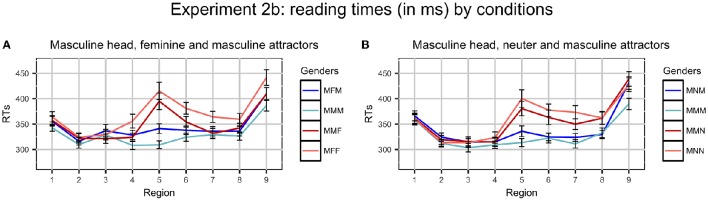
**Plots of mean RTs (in ms) by conditions in Experiment 2b**. Error bars represent standard errors of the means. Regions: NP_1_ (1) - preposition (2) - NP_2_ (3) - copula *byt'* (4) - Adj/Part (5) - spillover (6–9). Ungrammatical conditions are red, grammatical ones are blue. Conditions where the gender of the attractor and the predicate coincide (for example, MMM and MFF) have dark colors, conditions where they do not (for example, MFM and MMF) have light colors. **(A)** Masculine head, feminine and masculine attractors, **(B)** Masculine head, neuter and masculine attractors.

After three participants were excluded, on average, 1.5% RTs were excluded as outliers (never more than 3.1% per region and condition). Average RTs per region in different conditions are presented in Figure 2.

2 x 2 Repeated Measures ANOVAs with grammaticality and gender match as factors were used to analyze RTs, as in Experiment 2a. Significant results were found only in regions 5 (adjective/participle) and 6–7 (spillover regions). They are presented in Table 8.

**Table 8:** **Results of the analysis for Experiment 2b**.

**Conditions**	**Region**	**Factors**	**df**	**MS_*effect*_**	**F_1_**	***p***	**df**	**MS_*effect*_**	**F_2_**	***p***
MM vs. MF	5	Gram	**1.31**	**231038.53**	**17.55**	<**0.01**	**1.15**	**104660.34**	**15.92**	<**0.01**
		GenMatch	**1.31**	**19014.94**	**16.92**	<**0.01**	1.15	9218.4	0.93	0.35
		Gram ^*^ GenMatch	1.31	1974.28	0.66	0.42	1.15	887.3	0.13	0.72
	6	Gram	**1.31**	**42715.99**	**39.18**	<**0.01**	**1.15**	**23558.41**	**10.52**	**0.01**
		GenMatch	**1.31**	**11614.79**	**9.32**	**0.01**	1.15	6878.63	1.88	0.19
		Gram ^*^ GenMatch	1.31	1445.20	1.07	0.31	1.15	688.41	0.22	0.65
	7	Gram	**1.31**	**9604.98**	**12.30**	<**0.01**	1.15	4761	1.46	0.25
		GenMatch	**1.31**	**11881.11**	**20.57**	<**0.01**	1.15	7267.56	2.65	0.12
		Gram ^*^ GenMatch	1.31	4900.50	2.89	0.10	1.15	3825.42	1.19	0.29
MM vs. MN	5	Gram	**1.31**	**137333.85**	**33.02**	<**0.01**	**1.15**	**79129.69**	**10.12**	**0.01**
		GenMatch	1.31	14897.54	3.58	0.07	1.15	10686.39	1.66	0.22
		Gram ^*^ GenMatch	1.31	10.64	<0.01	0.96	1.15	129.96	0.02	0.89
	6	Gram	**1.31**	**75044.22**	**14.43**	<**0.01**	**1.15**	**36864.00**	**8.80**	**0.01**
		GenMatch	1.31	3404.16	1.34	0.26	1.15	1161.11	0.37	0.56
		Gram ^*^ GenMatch	1.31	2087.39	0.88	0.36	1.15	704.9	0.12	0.73
	7	Gram	**1.31**	**57868.02**	**23.86**	<**0.01**	**1.15**	**32761.00**	**8.15**	**0.01**
		GenMatch	**1.31**	**10235.23**	**7.27**	**0.01**	1.15	5016.18	1.25	0.28
		Gram ^*^ GenMatch	1.31	1362.42	0.53	0.47	1.15	419.23	0.1	0.76

*Analyses with p ≤ 0.05 are shown in bold*.

#### 4.4.1. Masculine head, feminine attractor

The main effect of Grammaticality was significant in analysis by subjects and by items in regions 5–6, and only in analysis by subjects in region 7. This reflects the fact that ungrammatical sentences were read slower than grammatical ones. The main effect of Gender Match was significant only in analysis by subjects in regions 5–7. The interaction between Grammaticality and Gender Match was not significant in any region.

#### 4.4.2. Masculine head, neuter attractor

The results were almost the same as in the other set of conditions. The main effect of Grammaticality was significant in regions 5–7. The main effect of Gender Match was significant only in analysis by subjects in regions 5–7. The interaction between the factors never reached significance.

### 4.5. Discussion

The results of this experiment show that the basic finding from Experiment 2a holds: there is no evidence for agreement attraction in the sentences with M heads. The plots of the data also suggest that the unbalanced frequencies in Experiment 2a had some influence on reading times. In Experiment 2b, where this confounding factor was excluded, two ungrammatical and two grammatical conditions pattern more closely together within each condition set. Still, the conditions where the genders of heads and attractors are mismatched have longer RTs.

Notably, this difference in RTs is not an instance of ungrammaticality illusion, since it is observed in both grammatical and ungrammatical conditions. In case of illusions, a different pattern would be expected: gender mismatch between the head and the attractor should increase RTs in grammatical conditions and decrease RTs in ungrammatical ones. Rather, it can be suggested that gender mismatch carries some processing cost in the sentences with M heads. In any case, our data do not allow for strong claims: the main effect Gender Match is significant in by subjects analysis in regions 5–7, but never reaches significance in by items analysis.

Since the outcome of comprehension experiments was not parallel to the results of Experiment 1 and earlier experiments on Slovak (Badecker and Kuminiak, 2007), we decided to look at the remaining combinations of head and attractor genders in Experiment 3 before suggesting an explanation.

## 5. Experiment 3

In this experiment, we studied sentences with N heads and N, F, and M attractors and sentences with F heads and F, N, and M attractors in comprehension. NF and FN combinations have not been examined before, and we added M attractors to be able to compare sentences with all possible attractors.

### 5.1. Participants

Thirty-nine native Russian speakers (22 female, 17 male) took part in the experiment. Ages ranged from 19 to 40 (mean age 25.4, *SD* 6.4).

### 5.2. Materials

We constructed 36 sets of stimuli according to the same schema as in Experiments 2a and 2b and observing the same constraints. Half of the sets had F head nouns and the other half had N head nouns. In all sets, we used M, N, and F attractors. Their frequency was closely matched inside the three groups of conditions, as Table 9 shows. Half of the predicates were grammatical, and half were not. As a result, every target sentence appeared in six conditions: NNN, NNF, NMN, NMM, NFN, NFF for the sentences with N heads and FFF, FFN, FMF, FMM, FNF, FNN for the sentences with F heads. Thus, out of all possible combinations of head, attractor and predicate genders, we did not use NNM and FFM. We decided to do so to keep the number of grammatical and ungrammatical conditions equal and sacrificed two conditions without any potential for agreement attraction that we have already looked at in Experiment 2a. Additionally, we used 100 fillers from Experiment 2a. Experimental sentences were distributed into six experimental lists, with factors counterbalanced. As a result, we had 136 sentences per list (18 ungrammatical and 118 grammatical), making the grammatical-to-ungrammatical ratio 6.6:1.

**Table 9:** **Frequencies of the attractors used in Experiment 3 (in ipm, or instances per million)**.

**Head gender**	**Attractor gender**	**Mean attractor freq (ipm)**
F	F	83.2
	M	76.6
	N	79.8
N	F	92.1
	M	92.8
	N	95.7

### 5.3. Procedure

The procedure was the same as in Experiments 2a and 2b. An experimental session lasted around 11 min.

### 5.4. Results

We analyzed participants' question-answering accuracy and reading times. The data from three participants were discarded because they had <75% accuracy in comprehension questions. As a result, we had 36 participants, six for each experimental list. None of them made more than two mistakes when answering questions to target sentences (i.e., 12.5% at most).

**Figure 3:**
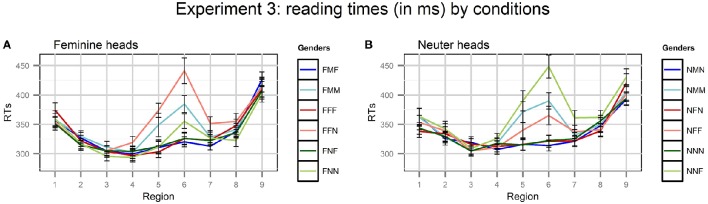
**Plots of mean RTs (in ms) by conditions in Experiment 3**. Error bars represent standard errors of the means. Regions: NP_1_ (1) - preposition (2) - NP_2_ (3) - copula *byt'* (4) - Adj/Part (5) - spillover (6–9). The conditions with M attractors are blue, with F attractors - red, with N attractors - green. Dark colors indicate grammatical conditions, light colors - ungrammatical conditions. **(A)** Feminine heads, **(B)** Neuter heads.

As in the previous experiments, reading times that exceeded a threshold of 2.5 standard deviations, by region and condition, were excluded. In total, 1.8% of the data were excluded (never more than 3.7% per region and condition). Average RTs per region in different conditions are presented in Figure 3 (notice that coloring conventions are different from the previous plots).

In Experiments 2a and 2b, we observed agreement attraction for some combinations of head and attractor genders (FM and NM), but not for the others (MF and MN). So the first question we asked in this experiment was whether there would be agreement attraction in NF and FN combinations. If the answer was yes, we were going to compare N and F attractors to M attractors. To answer the first question, we took two groups of conditions: FFF, FFN, FNF, FNN and NNN, NNF, NFN, NFF, and analyzed RTs using 2 x 2 Repeated Measures ANOVAs with grammaticality and gender match as factors, as in the previous experiments. Significant results were found only in regions 5 (adjective or participle) and 6–7 (a spillover region). They are presented in Table 10.

**Table 10:** **Results of the analysis for Experiment 3**.

**Conditions**	**Region**	**Factor**	**df**	**MS_*effect*_**	**F_1_**	***p***	**df**	**MS_*effect*_**	**F_2_**	***p***
FF vs. FN	5	Gram	**1.35**	**42202.85**	**13.54**	<**0.01**	**1.17**	**27667.36**	**14.05**	<**0.01**
		GenMatch	**1.35**	**24964.00**	**8.73**	**0.01**	1.17	14489.69	3.89	0.07
		GenMatch ^*^ Gram	**1.35**	**45411.61**	**12.98**	<**0.01**	1.17	13689.61	3.50	0.08
	6	Gram	**1.35**	**187272.56**	**17.49**	<**0.01**	**1.17**	**89662.01**	**12.62**	<**0.01**
		GenMatch	**1.35**	**67487.38**	**20.50**	<**0.01**	1.17	24857.07	2.33	0.15
		GenMatch ^*^ Gram	**1.35**	**65732.41**	**11.47**	<**0.01**	**1.17**	**40945.68**	**7.26**	**0.02**
NN vs. NF	5	Gram	**1.35**	**85176.42**	**23.48**	<**0.01**	**1.17**	**41103.22**	**7.33**	**0.02**
		GenMatch	**1,35**	**20168.73**	**5.43**	**0.03**	1.17	6258.94	1.05	0.32
		GenMatch ^*^ Gram	**1.35**	**25434.93**	**5.58**	**0.02**	1.17	7525.60	2.48	0.13
	6	Gram	**1.35**	**284248.92**	**32.88**	<**0.01**	**1.17**	**136808.09**	**41.17**	<**0.01**
		GenMatch	**1.35**	**79120.31**	**12.49**	<**0.01**	1.17	25233.81	3.98	0.06
		GenMatch ^*^ Gram	**1.35**	**76130.01**	**7.65**	**0.01**	**1.17**	**29658.30**	**4.78**	**0.04**

*Analyses with p ≤ 0.05 are shown in bold*.

#### 5.4.1. Feminine head, neuter attractor

The main effect of Grammaticality was significant in analysis by subjects and by items in regions 5–6. This reflects the fact that ungrammatical sentences were read slower than grammatical ones. The main effect of Gender Match was significant only in analysis by subjects in regions 5–6. The interaction of Grammaticality and Gender Match was significant in analysis by subjects and by items in region 6 and only in analysis by subjects in region 5. Ungrammatical sentences were read faster if the head and the attractor were mismatched in gender (i.e., in the FNN condition compared to the FFN condition). This is the classical attraction pattern, also known as a grammaticality illusion. At the same time, there are no differences between grammatical conditions, i.e., no evidence of ungrammaticality illusions was found.

#### 5.4.2. Neuter head, feminine attractor

The results were the same as in the other set of conditions. Thus, the answer to our first experimental question was positive, so we proceeded to compare the size of the attraction effect for attractors of different genders. We compared two groups of conditions: FNF, FNN, FMF, FMM and NFN, NFF, NMN, NMM. We used 2 x 2 Repeated Measures ANOVAs with grammaticality and attractor gender as factors. Only the main effect of Grammaticality in region 6 was statistically significant [for conditions with *F* heads, *F*_1_(1, 35) = 19.31, *p* <0.01, *MS*_*effect*_ = 86064.00; *F*_2_(1, 17) = 10.17, *p* = 0.01, *MS*_*effect*_ = 24457.35; for conditions with N heads, *F*_1_(1, 35) = 55.80, *p* <0.01, *MS*_*effect*_ = 126973.44; *F*_2_(1, 17) = 7.32, *p* = 0.02, *MS*_*effect*_ = 52915.47]. The main effect of Attractor Gender or the interaction between the factors were not significant in any region.

### 5.5. Discussion

Let us summarize the results of Experiments 2a, 2b, and 3. Gender agreement attraction was observed with F heads and M or N attractors and with N heads and M or F attractors, but not with M heads and F or N attractors. This leads us to the conclusion that attraction depends primarily on the features of the head rather than on the features of the attractor. If the features of the attractor played an additional role, ungrammatical sentences with M attractors would be read *faster* than ungrammatical sentences with other attractors. However, when we compared sentences with F heads and N or M attractors and sentences with N heads and F or M attractors, the Attractor Gender or the interaction between this factor and Grammaticality never reached significance, and average RTs even showed the opposite pattern: they were *longer* in the ungrammatical sentences with M attractors. This goes against the assumptions entertained in the absolute majority of previous agreement attraction studies, so a detailed analysis of this result will be presented in the General Discussion Section.

## 6. General discussion

In this paper we reported four experiments on gender agreement attraction in Russian. We observed attraction effects both in production and in comprehension. Badecker and Kuminiak (2007) is the only previous production study where gender agreement attraction was examined in a language with three genders (Lorimor et al., 2008 elicited very few gender errors in their experiments on Russian). In this paper, we replicated one of Badecker and Kuminiak's experiments and conducted the first comprehension experiments analyzing attraction with non-binary features.

Two outcomes of our experiments can be identified as the most important. Firstly, our results suggest that gender attraction works differently in production and comprehension. This does not agree with previous studies of number agreement attraction, in which production and comprehension results were largely parallel: only the combination of a singular head and a plural attractor triggered attraction. Secondly, our reading experiments suggest that the features of the head, rather than the features of the attractor are crucial to determine the pattern of agreement attraction, while the absolute majority of previous agreement attraction studies rely on the opposite assumption.

### 6.1. Overview of experimental findings

In our comprehension experiments, attraction was observed in some combinations of head and attractor genders, but not in the others, while in the production experiment, all combinations exhibited attraction, only to a different extent. We will first consider production results, and then comprehension findings. The outcome of the production study was similar to the results of the first experiment conducted by Badecker and Kuminiak (2007): there were more errors with MF subjects than with FM subjects and with NM subjects than with MN subjects. Both differences were statistically significant in the Slovak study, while in our experiment, only the first one was.

Badecker and Kuminiak ran an additional experiment comparing NF and NM preambles and found that the error rates in these conditions were roughly the same. They claim that this pattern can be explained only in an optimality-theoretic framework where markedness effects are by definition relational. We believe that this is not the case. Given the impressive body of literature on number and gender features, we do not think that we can select a particular approach based on experimental data without a detailed consideration of other arguments. So we chose two models that have been applied to Russian to demonstrate that they are also compatible with the pattern described by Badecker and Kuminiak and may be better suited to explain other findings we reported.

In Kramer (2015), F is encoded as [+FEM], M is [−FEM] and N corresponds to no gender features. When zero and non-zero feature values are compared, the latter are marked, and it can be argued that for this comparison, it is not important whether non-zero values are plus or minus. Therefore, the same error rates are observed with NF and NM preambles. When non-zero values are compared, plus values are more marked. In Nevins (2011), F is [+FEM], [−MASC], M is [−FEM], [+MASC] and N is [−FEM], [−MASC]. N is less marked than M and F because it contains only minus values, while M and F both contain one plus value. But when we compare F and M directly, it can be argued that feature hierarchy becomes important. [FEM] is standardly assumed to be lower than [MASC], so F is more marked than M.

Now let us focus on another property of production findings from Slovak and Russian that is not discussed by Badecker and Kuminiak (2007), but seems crucial to us. In case of gender agreement, attraction errors are produced with all preambles in which the genders of the head and the attractor are mismatched, while in case of number agreement, errors are virtually absent with plural heads and singular attractors. One way to capture this would be to assume that all genders are marked by some feature combinations, as Nevins (2011) suggests, while singular corresponds to no number features.

Another important problem is the difference between experimental findings from Slovak and Russian on the one hand and Romance languages on the other. In Russian and Slovak, more errors are produced with MF preambles than with FM preambles, while in Spanish and French the situation is the opposite. Badecker and Kuminiak (2007) do not comment on this discrepancy, and we cannot offer any explanation for it so far. We can only note that the pattern observed in Slovak and Russian is similar to what we see with number: more errors when the head is less marked than the attractor.

Now let us turn to comprehension experiments. Attraction was observed in NMM, NFF, FMM, and FNN conditions, but not in MFF and MNN conditions. As we already noted, this indicates that features of the heads rather than features of the attractors play a crucial role for attraction. Before discussing this finding in the next section, we want to make two important observations. Firstly, the M gender exhibits a different pattern from the F and N genders. This can hardly be attributed to feature markedness: N is the grammatical default in Russian, and the psycholinguistic relevance of this fact is confirmed by the production data discussed above. We will explore alternative explanations below. Secondly, no ungrammaticality illusions (differences between grammatical conditions depending on whether the head and the attractor have matched or mismatched gender features) were detected in our experiments, which lends further support to the retrieval approach to agreement attraction.

### 6.2. The role of head and attractor features in attraction

In the literature on agreement attraction, the presence or absence of the effect is traditionally associated with the features of the attractor. There are at least two reasons for this. Firstly, experimental findings suggest that *some* properties of attractors do influence attraction effects [e.g., as we discussed in the introduction, Hartsuiker et al. (2003) showed that the incidence of agreement errors was much higher when attractors were formally similar to nominative plural forms]. The second reason is tradition: the first proposed account of agreement attraction relied on feature percolation, which means focusing exclusively on the attractor whose features can erroneously spread upwards.

The assumption that the features of the attractor are crucial has been maintained in the more recent retrieval account. However, it is important to realize that in this account the properties of the head can influence the agreement process as well. For example, to explain the plural markedness effect, it is traditionally assumed that singular nouns are not marked for number, and “the system is biased to return explicitly number marked constituents” (Wagers et al., 2009, p. 233), therefore plural attractors can easily be retrieved, while singular ones almost never are. But another interpretation is possible: the plural feature makes the heads easier to retrieve and thus more stable, less prone to attraction errors. This is why attraction in the plural-singular configurations is virtually non-existent. On the other hand, the retrieval of singular heads is prone to error, hence the abundance of errors in singular-plural configurations^11^.

While we look at binary features or at the cases where attraction is observed in all feature combinations (as in production experiments on Slovak and Russian), we can only use indirect evidence to estimate the contribution of head and attractor features to the agreement process. Our reading experiments allow for the first direct comparison and show that at least in comprehension, the features of heads, not attractors play the crucial role. We observed attraction with attractors of all three genders, but only with N and F heads. The gender of the attractor did not even influence the size of the effect. These results suggest that the gender of the attractor has very little or no influence on its chances to be retrieved (it should only match the gender of the incorrect verb form).

Notably, Julie Franck expressed similar ideas in a recent talk (Franck, 2015). The first part of the talk was dedicated to summarizing existing data on agreement attraction. Franck adopted the retrieval approach for production and comprehension and identified the following groups of factors that can lead to attraction: semantic factors (primarily related to the conceptual numerosity of the subject NP), stability of the head's features, accessibility of the attractor (defined by its structural position) and similarity between the head and the attractor. Discussing stability of the head's features Franck examined asymmetries between feature values, morphophonological and semantic influences.

Franck's reexamination of attraction phenomena was driven by the findings on morphophonology (other data she considered could be accounted for in the old models). As we noted in the introduction, studies on several languages demonstrated that number and gender agreement attraction errors are less frequent when heads have regular inflections, but this plays no role for attractors (e.g., Bock and Eberhard, 1993; Vigliocco et al., 1995; Vigliocco and Zilli, 1999; Franck et al., 2008). For attractors, only morphological ambiguity making them more similar to a subject is important (e.g., Hartsuiker et al., 2003; Badecker and Kuminiak, 2007)^12^. This led Franck to conclude that the features of the head are crucial, and she reanalyzed existing data according to this idea. She argued that features that have a semantic correlate are more resistant to attraction (for example, Vigliocco and Franck, 1999 observed lower error rates when heads had conceptual rather than purely grammatical gender) and that the same is true for marked feature values. The latter conclusion was based on number agreement attraction findings and on the results of Badecker and Kuminiak's and our production experiments.

Thus, the findings summarized by Franck and the outcome of our reading experiments point into the same direction, but we still have to explain the difference between our comprehension and production results. Of course, to make definitive conclusions, it would be great to have data from several languages (for example, comprehension data from Slovak), but let us suggest several hypotheses based on existing findings. Our reading experiments strongly indicate that M heads are resistant to attraction, while N and F heads are not. The data from production experiments on Russian and Slovak are open to several interpretations because attraction was observed in all head-attractor combinations with mismatched genders. Therefore, we assume that M heads in general are the most stable ones and the least prone to attraction, and production data need an independent explanation. This assumption is supported by independent evidence: several production experiments on number agreement attraction in Russian reported by Nicol and Wilson (1999) and Yanovich and Fedorova (2006) demonstrated that the incidence of number errors depends on the gender of the head noun. Errors arise most often with N heads and least often with M ones.

If our assumption is on the right track, M heads and plural heads exhibit similar properties in comprehension. But why should they do so, given that M features are neither the most marked nor the least marked in Russian? Let us come back to the idea expressed in the previous subsection: number is privatively marked (i.e., singular nouns have no number features), while gender is not (all nouns have some gender features with plus and minus values). We hypothesize that with privative features, the non-zero value is the most stable, while with non-privative features, where all values are non-zero, other considerations come into the picture. We are reluctant to appeal to frequency, but maybe it plays a role that M gender vastly outnumbers F and N in Russian. In any case, our data indicate that that there is no straightforward relation between feature markedness and stability. The next subsection considers some differences between comprehension and production and how these differences could explain our results.

### 6.3. Differences between production and comprehension

Based on parallel results from number agreement attraction experiments most authors assume that the same mechanisms underlie attraction in production and comprehension. The opposite view has been recently advocated by Tanner et al. (2014). They claim that the mechanisms responsible for attraction in comprehension are a subset of those involved in production. In particular, they argue that attraction in comprehension is due to retrieval interference, while attraction in production is best described by the representational account, namely, by the Marking and Morphing model (Eberhard et al., 2005), although retrieval interference is also present.

As we noted above, the Marking and Morphing model is incompatible with gender agreement attraction. We believe that the core mechanism underlying number and gender agreement attraction in production is the same, so we opt for the retrieval approach. Evidently, in case of number, semantic factors influence agreement, and it is expected that their influence is much more readily detected in production than in comprehension: in production, we start with the conceptual structure, while in comprehension, it is our goal. Vigliocco and Franck (1999) demonstrated that gender agreement attraction errors are less frequent when head nouns have conceptual, rather than purely grammatical gender. So semantic factors also play a role here, but, given the relevant distinctions^13^ between number and gender, this role is different: they mainly reduce the size of the effect. It would be very interesting to assess their influence on gender agreement attraction in comprehension: we expect that it should be much smaller, as in case of number agreement. Thus, the differences between production and comprehension noted by Tanner et al. (2014) may also be relevant for gender agreement, but the picture revealed by our experiments cannot be explained by them.

In the previous subsection we argued that agreement attraction patterns in comprehension are due to the fact that heads with plural features and M features are resistant to attraction, i.e., that during the retrieval process, they tend to be identified correctly, while the retrieval of heads with other features can be disturbed by attractors. Findings summarized by Franck (2015) show that the stability of head's features should also be relevant for agreement attraction in production. This is further confirmed by the results from Nicol and Wilson (1999) to Yanovich and Fedorova (2006) indicating that heads with M features are indeed more stable when we look at number agreement production in Russian. Based on these data, we would expect to see no errors in MF and MN conditions in production experiments on gender agreement, but this is not what we found.

To address this problem, we should specify in more detail how retrieval may work in comprehension and production. Wagers et al. (2009) who analyze comprehension show that the retrieval account has two versions that may be difficult to tease apart based on the current experimental data. On the one hand, cue-based retrieval may be initiated every time we deal with an agreeing verb. On the other hand, we may predict the features of the upcoming verb relying on the subject NP and initiate retrieval only when our predictions are not met. Both versions give roughly the same results if we assume that when the true subject matches all the cues, it is successfully retrieved in the absolute majority of cases. Then in both scenarios, problems are expected only when we encounter an incorrect verb form and the sentence contains an attractor a non-subject NP that matches the incorrectly specified feature of the verb.

We believe that two similar scenarios can also be distinguished for production: we can decide which features we need on an agreeing predicate while processing the subject or once we get to the predicate. Accordingly, retrieval might be initiated every time we deal with an agreeing predicate or only when a wrong verb form that does not match our predictions is spuriously generated. The models proposed by Solomon and Pearlmutter (2004) or by Badecker and Kuminiak (2007) instantiate the first scenario. For example, Solomon and Pearlmutter argue that attraction in production arises because two nouns, the head of the subject NP and the attractor, are simultaneously active in the syntactic structure, and a wrong agreement controller may be selected. However, we argue for the second scenario below.

To summarize, in comprehension, we construct the set of retrieval cues based on the verb form that is provided to us. As we demonstrated above, different versions of the account share this basic observation. If the first scenario is adopted for production (the features of the upcoming verb are predicted, and retrieval is initiated only when we spuriously generate a wrong verb form), the picture should be quite similar: the set of cues will be based on this form.

However, we do not believe that this scenario is the most plausible. In particular, it implies that we generate the subject NP with all its feature specifications before we turn to the verb. In reality, the process should be much more complicated. On the one hand, we cannot determine the case of an NP before we select the predicate (for example, experiencers may receive nominative, accusative, or dative case in Russian, depending on the verb, so it is impossible to plan a nominative NP having only some abstract V in mind). On the other hand, we cannot select some features of the verb form without looking at the subject.

This leads us to adopt the second scenario, in which the relevant features are retrieved at some point during the derivation, rather than predicted and then rechecked. Then we do expect certain differences between production and comprehension. Namely, under the second production scenario it is not the case that we look for an NP with a particular number or gender feature. Rather, we look for the values of number and gender features inside the subject NP. These features should belong to the head of this NP, but sometimes we spuriously pay attention to the features of other nouns. We hypothesize that feature markedness plays a role in this process, and this is what causes different outcomes in our production and comprehension experiments.

To explain how markedness effects may arise, let us summarize different factors that have been shown to play a role for retrieval. More stable head nouns have more chances to be retrieved than less stable ones. Structurally accessible attractors looking like subjects have more chances to be retrieved than the attractors without these characteristics. This is true both for production and for comprehension. And, independently of these factors, marked features have more chances to be retrieved. In comprehension, when we encounter a particular verb form and construct a set of retrieval cues based on it, different number or gender features do not compete with each other: we always look for a particular value. In production, we need to find the value of the gender feature of the subject NP, there is no value that is provided in advance, thus different values may enter the competition^14^. Thus, production involves competition and comprehension does not, therefore we can observe feature markedness effects in production, but not in comprehension. This is why production and comprehension results for gender agreement are different. We do not observe any differences in case of number agreement because plural is at the same time a more stable feature and a marked one. This is a very tentative hypothesis, so further experiments are necessary to test it or to suggest an alternative explanation for the observed asymmetry between production and comprehension findings.

## Author contributions

All authors listed have made substantial, direct and intellectual contribution to the work, and approved it for publication.

### Conflict of interest statement

The authors declare that the research was conducted in the absence of any commercial or financial relationships that could be construed as a potential conflict of interest.

## Footnotes

^1^Here and further, the following standard symbols are used: N, noun; NP, noun phrase; P, preposition; PP, prepositional phrase; V, verb; M, masculine gender; F, feminine; N, neuter.

^2^Notably, in semantics there is an ongoing debate whether singular or plural is the default (e.g., Sauerland et al., 2005; Farkas and de Swart, 2010).

^3^In production, looking for symmetric effects in ungrammatical and grammatical sentences is less straightforward. However, several authors suggested not only counting errors, but also measuring RTs during elicitation tasks (e.g., Staub, 2009, 2010; Brehm and Bock, 2013). They demonstrated that participants slow down when the subject contains a singular head and a plural attractor both when they eventually answer correctly and when they do not [to be precise, Staub observed this for the subjects containing a PP attractor, but not for the subjects contained within relative clauses, as in (1b)].

^4^In combinations like MFM the first letter shows the gender of the head, the second letter - the gender of the attractor, the third letter (if present) the gender of the predicate.

^5^Since acceptability ratings for some sentences vary from speaker to speaker, we do not mark any of the examples below with asterisks or question marks used to indicate ungrammaticality or marginal acceptability.

^6^Vigliocco and Franck (1999) demonstrated that the gender agreement error rate was lower when the gender of the head noun was conceptual, rather than purely grammatical, but we would not expect markedness patterns to be reversed in such cases.

^7^As we explained in the introduction, participles, adjectives, and nouns in predicates can appear either in nominative or in instrumental, and adjectives and participles also have short forms used only in predicates and inflected for gender and number, but not for case. Often only one variant is grammatical, but sometimes two or even three are, or one is fine, while the others are marginally acceptable. Meaning nuances associated with them can be very subtle. It will suffice to say that we chose instrumental forms because, unlike nominative and short forms, they suited all our stimuli. But, if the participants occasionally responded with nominative or short forms, we did not count this as a mistake.

^8^We opted for this design primarily to facilitate the comparison with B&K's study. In addition to that, when we were pretesting the experiment, we found that the experimental session was relatively short, but very intense because we prompted the participants to respond very fast. We concluded that making it 1.5 times longer to fully cross the two factors could make it too taxing.

^9^In half of the conditions, where the genders of the head and the attractor coincided, no agreement errors with attraction were possible, while in the other half of the conditions, these errors prevailed, but there were also agreement errors without attraction, as Table 3 shows. This is why we chose the chi-square test.

^10^The translation for all sentences is identical, so we only give it for the first one.

^11^Let us note that under this scenario an attractor can also be retrieved in a singular-singular configuration, but this will not provoke any agreement errors.

^12^Let us add that Badecker and Kuminiak (2007) demonstrated that ambiguity is important not only for attractors, but also for heads: if the form is ambiguous between nominative and accusative, the chances of the head to be retrieved are lower.

^13^In case of number, we can find many words that are formally singular, but denote plural entities, for example, nouns like *crowd* or heads of the phrases like *the label on the bottles* that have a distributive interpretation. Gender is usually semantically empty, and when it is not, the conceptual and formal gender typically coincide (and thus the former reinforces the latter). If they do not coincide, it never depends on the properties of modifiers, only on the noun itself (for example, *vrač* “doctor_*M*_” can refer both to a man and to a woman in Russian).

^14^In our production experiment, participants were provided with predicates in a particular form. Still, we also expect competition here because participants had to produce a correct form if the provided form was wrong, and to do so, they had to retrieve the subject NP and determine its gender.
