# Supplementary material for: Gender Agreement Attraction in Russian: Production and Comprehension Evidence
Source: Front Psychol. 2016 Nov 4;7:1651. doi: 10.3389/fpsyg.2016.01651 (PMC5095607; doi:10.3389/fpsyg.2016.01651)
Supplement: Supplementary file 1 [file DataSheet1.pdf]

Supplementary Material: Gender agreement attraction in Russian: production and comprehension evidence

Natalia Slioussar*, Anton Malko
*Correspondence:
Natalia Slioussar
slioussar@gmail.com

1 EXPERIMENT 1

Table 1: Experiment 1 stimuli

Preamble	Predicate
Okno v pole / dvor<br>window$_N$ to field$_N$ / yard$_M$	bylo otkrytym / byl otkrytym<br>was opened$_{N/M}$
Mesto pod radio / televizor<br>place$_N$ for radio$_N$ / TV-set$_M$	bylo svobodnym / byl svobodnym<br>was not.occupied$_{N/M}$
Vino pod mjaso / syr<br>wine$_N$ for meat$_N$ / cheese$_M$	bylo kislym / byl kislym<br>was sour$_{N/M}$
Upražnenie na čtenie / sčet<br>exercise$_N$ in reading$_N$ / counting$_M$	bylo trudnym / byl trudnym<br>was difficult$_{N/M}$
Vremja na zadanie / otdyx<br>time$_N$ for task$_N$ / rest$_M$	bylo nedostatočnym / byl nedostatočnym<br>was insufficient$_{N/M}$
Razrešenie na stroitel'stvo / snos<br>permit$_N$ for construction$_N$ / demolition$_M$	bylo nezakonnym / byl nezakonnym<br>was illegal$_{N/M}$
Pravo na sčast'e / otdyx<br>right$_N$ for happiness$_N$ / rest$_M$	bylo vseobščim / byl vseobščim<br>was universal$_{N/M}$
Pis'mo pro prikljućenie / poxod<br>letter$_N$ about adventure$_N$ / camping$_M$	bylo uvlekatel'nym / byl uvlekatel'nym<br>was interesting$_{N/M}$
Ranenie v plečo / život<br>wound$_N$ in shoulder$_N$ / belly$_M$	bylo legkim / byl legkim<br>was light$_{N/M}$
Pogruženie v more / okean<br>dive$_N$ in sea$_N$ / ocean$_M$	bylo dolgim / byl dolgim<br>was long$_{N/M}$

Table 1: Experiment 1 stimuli

**Preamble**	**Predicate**
Vozdejstvie na soznanie / razum<br>impact$_N$ on consciousness$_N$ / mind$_M$	bylo nezametnym / byl nezametnym<br>was imperceptible$_{N/M}$
Zajavlenie na uvol'nenie / otpusk<br>application$_N$ for job.quit$_N$ / leave$_M$	bylo formal'nym / byl formal'nym<br>was formal$_{N/M}$
Stakan pod čaj / moloko<br>cup$_M$ for tea$_M$ / milk$_N$	byl stekljannym / bylo stekljannym<br>was made.of.glass$_{M/N}$
Vyxod v les / pole<br>exit$_M$ to forest$_M$ / field$_N$	byl zabrošennym / bylo zabrošennym<br>was abandonned$_{M/N}$
Vzgljad na ekran / izobraženie<br>look$_M$ at screen$_M$ / image$_N$	byl beglym / bylo beglym<br>was cursory$_{M/N}$
Otvet na vopros / vozraženie<br>answer$_M$ to question$_M$ / objection$_N$	byl bystrym / bylo bystrym<br>was quick$_{M/N}$
Vid na zaliv / more<br>view$_M$ of gulf$_M$ / sea$_N$	byl vpečatljajuščim / bylo vpečatljajuščim<br>was impressive$_{M/N}$
Pryžok čerez zabor / ograždenie<br>jump$_M$ over fence$_M$ / hedge$_N$	byl neudačnym / bylo neudačnym<br>was not.successful$_{M/N}$
Udar o kosjak / derevo<br>hit$_M$ against doorjamb$_M$ / tree$_N$	byl sil'nym / bylo sil'nym<br>was strong$_{M/N}$
Test na intellekt / vnimanie<br>test$_M$ of intelligence$_M$ / attention$_N$	byl složnym / bylo složnym<br>was difficult$_{M/N}$
Bilet na spektakl' / predstavlenie<br>ticket$_M$ for play$_M$ / show$_N$	byl deševym / bylo deševym<br>was cheap$_{M/N}$
Bočonok pod rom / vino<br>barrel$_M$ for rhum$_M$ / wine$_N$	byl polnym / bylo polnym<br>was full$_{M/N}$
Talon na osmotr / obsledovanie<br>ticket$_M$ for examination$_M$ / examination$_N$	byl mjatym / bylo mjatym<br>was crumpled$_{M/N}$
Propusk v universitet / obščežitie<br>id.card$_M$ for university$_M$ / dormitory$_N$	byl nedejstvitel'nym / bylo nedejstvitel'nym<br>was invalid$_{M/N}$
Vystrel v vozdux / mišen'<br>shot$_M$ in air$_M$ / target$_F$	byl oglušitel'nym / byla oglušitel'noj<br>was defeaning$_{M/F}$
Udar v život / grud'	byl moščnym / byla moščnoj

Table 1: Experiment 1 stimuli

**Preamble**	**Predicate**
hit$_M$ in belly$_M$ / chest$_F$	was strong$_{M/F}$
Perevod na russkij / latyn'<br>translation$_M$ into Russian$_M$ / Latin$_F$	byl nepravil'nym / byla nepravil'noj<br>was wrong$_{M/F}$
Spros na metall / med'<br>demand$_M$ for metal$_M$ / copper$_F$	byl značitel'nym / byla značitel'noj<br>was significant$_{M/F}$
Nabaldašnik na posox / trost'<br>knob$_M$ on staff$_M$ / walking.stick$_F$	byl uvesistym / byla uvesistoj<br>was heavy$_{M/F}$
Laz pod zabor / izgorod'<br>hole$_M$ under fence$_M$ / hedge$_F$	byl uzkim / byla uzkoj<br>was narrow$_{M/F}$
Zakaz na tovar / detal'<br>order$_M$ for product$_M$ / spare.part$_F$	byl sročnym / byla sročnoj<br>was urgent$_{M/F}$
Recept na porošok / maz'<br>prescription$_M$ for powder$_M$ / ointment$_F$	byl prosročennym / byla prosročennoj<br>was outdated$_{M/F}$
Material na pidžak / vual'<br>fabric$_M$ for jacket$_M$ / veil$_F$	byl krasivym / byla krasivoj<br>was beautiful$_{M/F}$
Pretendent na tron / dolžnost'<br>contender$_M$ for throne$_M$ / position$_F$	byl samouverennym / byla samouverennoj<br>was self-assured$_{M/F}$
Vxod v teatr / cerkov'<br>entrance$_M$ to theater$_M$ / church$_F$	byl zakrytym / byla zakrytoj<br>was closed$_{M/F}$
Nalog na avtomobil' / sobstvennost'<br>tax$_M$ on car$_M$ / property$_F$	byl vysokim / byla vysokoj<br>was high$_{M/F}$
Ustanovka na effektivnost' / rezul'tat<br>directive$_F$ at effectiveness$_F$ / result$_M$	byla dejstvennoj / byl dejstvennym<br>was efficient$_{F/M}$
Nakidka na krovat' / divan<br>cover$_F$ for bed$_F$ / couch$_M$	byla raznocvetnoj / byl raznocvetnym<br>was multi-colored$_{F/M}$
Doroga na ploščad' / rynok<br>road$_F$ to plaza$_F$ / market$_M$	byla širokoj / byl širokim<br>was wide$_{F/M}$
Cena na sol' / saxar<br>price$_F$ for salt$_F$ / sugar$_M$	byla zapredel'noj / byl zapredel'nym<br>was exorbitant$_{F/M}$
Recenzija na povest' / roman<br>review$_F$ of story$_F$ / novel$_M$	byla razgromnoj / byl razgromnym<br>was scathing$_{F/M}$

Table 1: Experiment 1 stimuli

**Preamble**	**Predicate**
Reakcija na novost' / uprek<br>reaction$_F$ on news$_F$ / reproach$_M$	byla neadekvatnoj / byl neadekvatnym<br>was inadequate$_{F/M}$
Rasplata za podlost' / obman<br>retribution$_F$ for vileness$_F$ / lying$_M$	byla skoroj / byl skorym<br>was quick$_{F/M}$
Nadežda na spravedlivost' / uspex<br>hope$_F$ for justice$_F$ / success$_M$	byla pročnoj / byl pročnym<br>was solid$_{F/M}$
Proverka na pročnost' / intellekt<br>test$_F$ of stamina$_F$ / intelligence$_M$	byla utomitel'noj / byl utomitel'nym<br>was exhausting$_{F/M}$
Bor'ba za vlast' / prestol<br>struggle$_F$ for power$_F$ / throne$_M$	byla ožestočennoj / byl ožestočennym<br>was violent$_{F/M}$
Bitva za žizn' / gorod<br>fight$_F$ for life$_F$ / town$_M$	byla žestokoj / byl žestokim<br>was fierce$_{F/M}$
Plata za noč' / vxod<br>price$_F$ for night$_F$ / entrance$_M$	byla nevysokoj / byl nevysokim<br>was low$_{F/M}$

2 EXPERIMENT 2A

Table 2: Experiment 2a stimuli

Preamble	Predicate	Sent.continuation
Vystrel v vozdux / mišen'<br>shot$_M$ in air$_M$ / target$_F$	byl proizveden / byla proizvedena<br>was made$_{M/F}$	iz tabel'nogo oružija.<br>from assigned weapon.
Most čerez ovrag / propast'<br>bridge$_M$ over ravine$_M$ / precipice$_F$	byl sooružen / byla sooružena<br>was built$_{M/F}$	pered rešajuščim nastupleniem.<br>before the decisive attack.
Perevod na russkij / latyn'<br>translation$_M$ into Russian$_M$ / Latin$_F$	byl zakončen / byla zakončena<br>was finished$_{M/F}$	za pjatnadcat' minut.<br>in fifteen minutes
Spros na metall / med'<br>demand$_M$ for metall$_M$ / copper$_F$	byl razogret / byla razogreta<br>was heated up$_{M/F}$	iz-za nestabil'noj rynočnoj situacii.<br>becuase of unstable situation on the market.
Nabaldašnik na posox / trost'<br>knob$_M$ on stuff$_M$ / walking.stick$_F$	byl vyrezan / byla vyrezana<br>was carved$_{M/F}$	v vide golovy pudelja.<br>in the shape of a dog's head.
Laz pod zabor / izgorod'<br>hole$_M$ under fence$_M$ / hedge$_F$	byl zadelan / byla zadelana<br>was blocked$_{M/F}$	s pomošč'ju pary dosok.<br>by a couple of planks.
Zakaz na detal' / tovar<br>order$_M$ for spare.part$_F$ / goods$_M$	byl oformlen / byla oformlena<br>was completed$_{M/F}$	so vsej vozmožnoj pospešnost'ju.<br>as quickly as possible.
Recept na maz' / porošok<br>prescription$_M$ for ointment$_F$ / powder$_M$	byl vypisan / byla vypisana<br>was issued$_{M/F}$	na oficial'nom blanke polikliniki.<br>on the official hospital letterhead.
Material na vual' / pidžak<br>fabric$_M$ for veil$_F$ / jacket$_M$	byl priobreten / byla priobretena<br>was bought$_{M/F}$	v central'nom univermage goroda.<br>in the central town store.
Otbor na dolžnost' / konkurs<br>selection$_M$ for position$_F$ / competition$_M$	byl proveden / byla provedena<br>was organized$_{M/F}$	s narušeniem rossijskogo zakonodatel'stva.<br>with violations of Russian laws.
Vxod v cerkov' / teatr<br>entrance$_M$ to church$_F$ / theater$_M$	byl zakryt / byla zakryta<br>was closed$_{M/F}$	iz-za nedavno načavšegosja remonta.<br>because of recently started repairs.
Nalog na sobstvennost' / avtomobil'<br>tax$_M$ on property$_F$ / car$_M$	byl uveličen / byla uveličena<br>was increased$_{M/F}$	posle martovskogo zasedanija pravitel'stva.<br>after the govrnement session in March.
Ustanovka na effektivnost' / rezul'tat<br>directive$_F$ at effectiveness$_F$ / result$_M$	byla sformulirovana / byl sformulirovan<br>was formulated$_{F/M}$	v samom načale soveščanija.<br>in the very beginning of the meeting.

Table 2: Experiment 2a stimuli

**Preamble**	**Predicate**	**Sent.continuation**
Nakidka na krovat' / divan<br>cover$_F$ for bed$_F$ / couch$_M$	byla rasšita / byl rasšit<br>was embroidered$_{F/M}$	v roskošnom vostočnom stile.<br>in splendid oriental style.
Doroga na ploščad' / rynok<br>road$_F$ to plaza$_F$ / market$_M$	byla razmyta / byl razmyt<br>was eroded$_{F/M}$	posle nedavnix prolivnyx doždej.<br>after recent heavy rains.
Cena na sol' / saxar<br>price$_F$ for salt$_F$ / sugar$_M$	byla povyšena / byl povyšen<br>was raised$_{F/M}$	po pričine istoščenija zapasov.<br>because of the exhaustion of the supplies.
Recenzija na povest' / rasskaz<br>review$_F$ of story$_F$ / short.story$_M$	byla napisana / byl napisan<br>was written$_{F/M}$	dlja izvestnogo literaturnogo žurnala.<br>for a famous literary magazine.
Reakcija na novost' / uprek<br>reaction$_F$ on news$_F$ / reproach$_M$	byla predskazuema / byl predskazuem<br>was predictable$_{F/M}$	iz-za carivšego vokrug unynija.<br>since everybody around was feeling down.
Rasplata za obman / podlost'<br>retribution$_F$ for lying$_M$ / vileness$_F$	byla bespoščadna / byl bespoščaden<br>was ruthless$_{F/M}$	iz-za krutogo nrava gercoga.<br>becuase of the bad temper of the duke.
Nadežda na uspex / spravedlivost'<br>hope$_F$ for success$_M$ / justice$_F$	byla nepokolebima / byl nepokolebim<br>was solid$_{F/M}$	na protjaženii vsego processa.<br>during the whole trial.
Žaloba na stress / ustalost'<br>complaint$_F$ about stress$_M$ / tiredness$_F$	byla vosprinjata / byl vosprinjat<br>was taken$_{F/M}$	okružajuščimi kak projavlenie slabosti.<br>as a sign of weakness.
Bor'ba za prestol / vlast'<br>struggle$_F$ for throne$_M$ / power$_F$	byla razvjazana / byl razvjazan<br>was unleashed$_{F/M}$	posle neudačnogo dvorcovogo perevorota.<br>after an unsuccessful coup d'etat.
Blagodarnost' za sovet / pomošč'<br>gratitude$_F$ for advice$_M$ / help$_F$	byla proiznesena / byl proiznesen<br>was uttered$_{F/M}$	s ploxo skryvaemym razdraženiem.<br>with barely hidden irritation.
Plata za obed / noč'<br>price$_F$ for dinner$_M$ / night$_F$	byla nevelika / byl nevelik<br>was low$_{F/M}$	dlja studentov gosudarstvennyx vuzov.<br>for the students of public universities.
Stakan pod čaj / moloko<br>cup$_M$ for tea$_M$ / milk$_N$	byl sdelan / bylo sdelano<br>was made$_{M/N}$	iz tolstogo matovogo stekla.<br>from a thick opaque glass.
Vezd v zamok / imenie<br>entrance$_M$ to castle$_M$ / estate$_N$	byl perekryt / bylo perekryto<br>was abandonned$_{M/N}$	iz-za zatjanuvšixsja dorožnyx rabot.<br>because of long-drawn road work.

Table 2: Experiment 2a stimuli

**Preamble**	**Predicate**	**Sent.continuation**
Vzgljad na risunok / izobraženie<br>look$_M$ at drawing$_M$ / picture$_N$	byl brošen / bylo brošeno<br>was made$_{M/N}$	iz-pod koketlivo opuščennyx resnic.<br>from under the lowered eyelashes.
Otvet na vopros / vozraženie<br>answer$_M$ to question$_M$ / objection$_N$	byl ocenen / bylo oceneno<br>was appreciated$_{M/N}$	po dostoinstvu vsemi ekspertami.<br>by all the experts.
Vid na zaliv / ozero<br>view$_M$ of gulf$_M$ / lake$_N$	byl isporčen / bylo isporčeno<br>was spoiled$_{M/N}$	iz-za neokončennoj strojki.<br>because of an unfinished construction.
Pryžok čerez bar'er / prepjactvie<br>jump$_M$ over barrier$_M$ / obstacle$_N$	byl vypolnen / bylo vypolneno<br>was performed$_{M/N}$	bez pomarok vsemi sporcmenami.<br>perfectly by all the sportsmen.
Udar o derevo / kamen'<br>hit$_M$ against tree$_N$ / stone$_M$	byl neizbežen / bylo neizbežno<br>was inevitable$_{M/N}$	iz-za sil'nogo zanosa avtomobilja.<br>because of the car skidding.
Test na vnimanie / intellekt<br>test$_M$ of attention$_N$ / intelligence$_M$	byl projden / bylo projdeno<br>was completed$_{M/N}$	bez ser'eznyx ošibok.<br>without serious errors.
Bilet na predstavlenie / spektakl'<br>ticket$_M$ for show$_N$ / play$_M$	byl dešev / bylo deševo<br>was cheap$_{M/N}$	dlja učaščixsja mladšix klassov.<br>for the primary school students.
Bočonok pod vino / portvejn<br>barrel$_M$ for wine$_N$ / port$_M$	byl napolnen / bylo napolneno<br>was full$_{M/N}$	do kraev prostoj vodoj.<br>to the rim with simple water.
Talon na obsledovanie / osmotr<br>ticket$_M$ for examination$_N$ / examination$_M$	byl izmjat / bylo izmjato<br>was crumpled$_{M/N}$	iz-za sil'nogo volnenija pacienta.<br>because of the patient's agitation.
Propusk v obščežitie / universitet<br>id.card$_M$ for dormitory$_N$ / iuniversity$_M$	byl izgotovlen / bylo izgotovleno<br>was made$_{M/N}$	iz beloj blestjaščej plastmassy.<br>from white glossy plastic.
Okno v pole / dvor<br>window$_N$ to field$_N$ / yard$_M$	bylo otkryto / byl otkryt<br>was opened$_{N/M}$	iz-za stojavšej vnutri duxoty.<br>because it was stuffy inside.
Mesto pod radio / televizor<br>place$_N$ for radio$_N$ / TV-set$_M$	bylo vydeleno / byl vydelen<br>was chosen$_{N/M}$	na babuškinom komode.<br>on the grand-mother's hest of drawers.
Vino pod mjaso / syr<br>wine$_N$ for meat$_N$ / čeese$_M$	bylo kupleno / byl kuplen<br>was bought$_{N/M}$	v malen'kom magazinčike.<br>in a little shop.
Upražnenie na čtenie / sčet<br>exercise$_N$ in reading$_N$ / counting$_M$	bylo sostavleno / byl sostavlen<br>was developped$_{N/M}$	s učetom vozrastnyx osobennostej.<br>with age-specific characteristics in mind.

Table 2: Experiment 2a stimuli

**Preamble**	**Predicate**	**Sent.continuation**
Vremja na zadanie / otdyx<br>time$_N$ for task$_N$ / rest$_M$	bylo ograničeno / byl ograničen<br>was limited$_{N/M}$	v silu složivšixsja obstojatel'stv.<br>because of the circumstances.
Razrešenie na stroitel'stvo / remont<br>permit$_N$ for construction$_N$ / repairs$_M$	bylo podpisano / byl podpisan<br>was signed$_{N/M}$	posle burnogo obsuždenija proekta.<br>after long discussions of the project.
Pravo na vybor / sčast'e<br>right$_N$ for choice$_M$ / happiness$_N$	bylo opredeleno / byl opredelen<br>was defined$_{N/M}$	kak naše neotemlemoe dostojanie.<br>as our inalienable property.
Pis'mo pro poxod / priključenie<br>letter$_N$ about camping$_M$ / adventure$_N$	bylo otpravleno / byl otpravlen<br>was sent$_{N/M}$	iz gluxoj sibirskoj derevuški.<br>from a remote village in Syberia.
Ranenie v život / plečo<br>wound$_N$ in belly$_M$ / shoulder$_N$	bylo polučeno / byl polučen<br>was received$_{N/M}$	v xode krovoprolitnogo sraženija.<br>during a slaughterous combat.
Pogruženie v okean / more<br>dive$_N$ in ocean$_M$ / sea$_N$	bylo otloženo / byl otložen<br>was postponed$_{N/M}$	iz-za vnezapno razygravšejsja buri.<br>because of a sudden storm.
Vozdejstvie na razum / soznanie<br>impact$_N$ on mind$_M$ / consciousness$_N$	bylo zametno / byl zameten<br>was noticeable$_{N/M}$	po izmenivšemusja povedeniju ispytuemogo.<br>from the changed behavior of the subject.
Zajavlenie na otpusk / uvol'nenie<br>application$_N$ for vacation$_M$ / job.quit$_N$	bylo rassmotreno / byl rassmotren<br>was processed$_{N/M}$	v tečenie dvux nedel'.<br>in two weeks.

3 EXPERIMENT 2B

Table 3: Experiment 2b stimuli

Preamble	Predicate	Sent.continuation
Povorot v poselok / step'<br>turn$_M$ to village$_M$ / steppe$_F$	byl viden / byla vidna<br>was visible$_{M/F}$	s veršiny vysokogo xolma.<br>from the top of a hill.
Štraf za obman / glupost'<br>fine$_M$ for fraud$_M$ / fooling.around$_F$	byl bol'šim / byla bol'šoj<br>was big$_{M/F}$	iz-za surovosti novogo direktora.<br>because of the harshness of the new director.
Fil'm pro podvig / ljubov'<br>movie$_M$ about feat$_M$ / love$_F$	byl snjat / byla snjata<br>was filmed$_{M/F}$	v sliškom pafosnoj manere.<br>with too much pathos.
Boj za aerodrom / krepost'<br>battle$_M$ for airfield$_M$ / forteress$_F$	byl perelomnym / byla perelomnoj<br>was critical$_{M/F}$	dlja xoda graždanskoj vojny.<br>for the course of the civil war.
Zakaz na tovar / mebel'<br>order$_M$ for goods$_M$ / furniture$_F$	byl vypolnen / byla vypolnena<br>was completed$_{M/F}$	s sobljudeniem ogovorennyx srokov.<br>in accordance with the deadlines.
Romans pro večer / grust'<br>song$_M$ about night$_M$ / sadness$_F$	byl populjaren / byla populjarna<br>was popular$_{M/F}$	sredi dam bal'zakovskogo vozrasta.<br>among middle-aged women.
Traktat pro razum / ličnost'<br>treatise$_M$ about reason$_M$ / personality$_F$	byl sostavlen / byla sostavlena<br>was composed$_{M/F}$	v načale srednix vekov.<br>in the early Middle Ages.
Kontejner pod gorox / fasol'<br>jar$_M$ for peas$_M$ / beans$_F$	byl zapolnen / byla zapolnena<br>was filled$_{M/F}$	počti do samyx kraev.<br>almost to the rim.
Otčet pro poxod / neprijatnost'<br>report$_M$ about camping.trip$_M$ / issue$_F$	byl otpravlen / byla otpravlena<br>was sent$_{M/F}$	iz dalekoj sibirskoj derevuški.<br>from a remote village in Syberia.
Vygovor za obman / podlost'<br>reprimand$_M$ for lying$_M$ / vileness$_F$	byl surovym / byla surovoj<br>was harsh$_{M/F}$	daže dlja byvalogo xuligana.<br>even for the tough bully.
Orden za podvig / smelost'<br>order$_M$ for feat$_M$ / courage$_F$	byl vručen / byla vručena<br>was handed$_{M/F}$	v xode toržestvennoj ceremonii.<br>at an official ceremony.
Pryžok na divan / krovat'<br>jump$_M$ on couch$_M$ / bed$_F$	byl rascenen / byla rascenena<br>was considered$_{M/F}$	kak narušenie pravil priličija.<br>a violation of decorum.
Trakt čerez poselok / step'<br>road$_M$ through village$_M$ / steppe$_F$	byl zabrošen / byla zabrošena<br>was abandonned$_{M/F}$	v konce prošlogo veka.<br>in the end of the last century.
Vystrel v stend / mišen'<br>shot$_M$ at stand$_M$ / target$_F$	byl proizveden / byla proizvedena<br>was performed$_{M/F}$	s nevidannoj prežde točnost'ju.<br>with an unheard-of accuracy

Table 3: Experiment 2b stimuli

**Preamble**	**Predicate**	**Sent.continuation**
Put' na vokzal / ploščad'<br>road$_M$ to train.station$_M$ / plaza$_F$	byl utomitel'nym / byla utomitel'noj<br>was exhausting$_{M/F}$	iz-za nevynosimo žarkogo solnca.<br>because of extermely hot weather.
Gonorar za perevod / povest'<br>honorarium$_M$ for translation$_M$ / short.story$_F$	byl vyplačen / byla vyplačena<br>was paid$_{M/F}$	posle neskol'kix mesjacev zaderžki.<br>after several months of delay.
Recept na porošok / lekarstvo<br>prescription$_M$ for powder$_M$ / drug$_N$	byl pomjat / bylo pomjato<br>was crumpled$_{M/N}$	iz-za častogo predjavlenija aptekarjam.<br>because of being used too often.
Šelk na narjad / plat'e<br>silk$_M$ for garment$_M$ / dress$_N$	byl priobreten / bylo priobreteno<br>was bought$_{M/N}$	v central'nom univermage goroda.<br>in the city central store.
Pretendent na prestol / gospodstvo<br>contender$_M$ for throne$_M$ / power$_N$	byl ubit / bylo ubito<br>was killed$_{M/N}$	v rezul'tate dvorcovogo perevorota.<br>during the coup d'etat.
Vxod v magazin / zdanie<br>entrance$_M$ to shop$_M$ / building$_N$	byl zakryt / bylo zakryto<br>was closed$_{M/N}$	iz-za nedavno načavšegosja remonta.<br>because of the recently started repairs.
Rasskaz pro kašel' / zdorov'e<br>story$_M$ about cough$_M$ / health$_N$	byl vyslušan / bylo vyslušano<br>was listened$_{M/N}$	vračom s bol'šim vnimaniem.<br>by the doctor with great attention.
Roman pro zagovor / dectvo<br>novel$_M$ about conspiracy$_M$ / childhood$_N$	byl opublikovan / bylo opublikovano<br>was published$_{M/N}$	v nebol'šom moskovskom izdatel'stve.<br>in a small publishing house in Moscow.
Šans na uspex / spasenie<br>chance$_M$ for success$_M$ / rescue$_N$	byl upuščen / bylo upuščeno<br>was missed$_{M/N}$	v samyj poslednij moment.<br>in the very last moment.
Most čerez potok / boloto<br>bridge$_M$ over torrent$_M$ / swamp$_N$	byl nenadežnym / bylo nenadežnym<br>was unstable$_{M/N}$	posle nedavnix prolivnyx doždej.<br>after recent heavy rains.
Spusk v tonnel' / ušček'e<br>descent$_M$ into tunnel$_M$ / canyon$_N$	byl osuščestvlen / bylo osuščestvleno<br>was carried.out$_{M/N}$	so vsemi vozmožnymi predostorožnostjami.<br>with every possible precaution.
Material na zabor / ograždenie<br>material$_M$ for fence$_M$ / fence$_N$	byl privezen / bylo privezeno<br>was brought$_{M/N}$	so sklada ucenennyx tovarov.<br>from the discount materials warehouse.
Otvet na uprek / soobščenie<br>answer$_M$ to reprimand$_M$ / message$_N$	byl predskazuemym / bylo predskazuemym<br>was predictable$_{M/N}$	iz-za carivšego vokrug unynija.<br>because everybody around was feeling down.

Table 3: Experiment 2b stimuli

Preamble	Predicate	Sent.continuation
Otzyv na roman / esse<br>review$_M$ on novel$_M$ / essay$_N$	byl napisan / bylo napisano<br>was written$_{M/N}$	dlja izvestnogo literaturnogo žurnala.<br>for a famous literary magazine.
Otpusk na dekabr' / leto<br>job.leave$_M$ for December$_M$ / summer$_N$	byl zaplanirovan / bylo zaplanirovano<br>was planned$_{M/N}$	do padenija kursa rublja.<br>before the fall of the rouble.
Spros na benzin / toplivo<br>demand$_M$ on gas$_M$ / fuel$_N$	byl povyšen / bylo povyšeno<br>was increased$_{M/N}$	po pričine istoščenija zapasov.<br>because of the exhaustion of the supplies.
Udar v život / plečo<br>hit$_M$ in belly$_M$ / shoulder$_N$	byl nanesen / bylo naneseno<br>was received$_{M/N}$	v xode zavjazavšejsja draki.<br>during the fight.
Test na sčet / čtenie<br>test$_M$ of counting$_M$ / reading$_N$	byl sostavlen / bylo sostavleno<br>was developped$_{M/N}$	s učetom vozrastnyx osobennostej.<br>with age-specific characteristics in mind.

4 EXPERIMENT 3

Table 4: Experiment 3 stimuli

Preamble	Predicate	Sent.continuation
Vino pod salat / dič' / mjaso<br>wine$_N$ for salad$_M$ / fowl$_F$ / meat$_N$	byl kuplen / byla kuplena / bylo kupleno<br>was bought$_{M/F/N}$	v magazinčike na okraine.<br>in a little shop in the outskirts.
Vlijanie na komitet / cerkov' / obščestvo<br>impact$_N$ on commitee$_M$ / church$_F$ / society$_N$	byl nezametnym / byla nezametnoj / bylo nezametnym<br>was not.noticeable$_{M/F/N}$	dlja neposvjaščennyx v zagovor.<br>for those who were not part of the conspiracy.
Vozdejstvie na razum / ličnost' / soznanie<br>influence$_N$ on mind$_M$ / personality$_F$ / consciousness$_N$	byl zameten / byla zametna / bylo zametno<br>was noticeable$_{M/F/N}$	po izmenivšemusja povedeniju ispytuemogo.<br>by the changed behavior of the subject.
Izvinenie za besporjadok / glupost' / xamstvo<br>apology$_N$ for clutter$_M$ / foolishness$_F$ / rudeness$_N$	byl prinesen / byla prinesena / bylo prineseno<br>was given$_{M/F/N}$	s ploxo skryvaemym razdraženiem.<br>with barely concealed irritation.
Kruževo na narjad / šal' / plat'e<br>lace$_N$ for garment$_M$ / shawl$_F$ / dress$_N$	byl priobreten / byla priobretena / bylo priobreteno<br>was bought$_{M/F/N}$	v central'nom univermage goroda.<br>in the central city store.

Table 4: Experiment 3 stimuli

Preamble	Predicate	Sent.continuation
Naznačenie na post / dolžnost' / rukovodstvo	byl vygodnym / byla vygodnoj / bylo vygodnym	dlja storonnikov pravjaščej dinastii.
appointment$_N$ to office$_M$ / position$_F$ / direction$_N$	was advantageous$_{M/F/N}$	for the supporters of the reigning dynasty.
Nakazanie za obman / podlost' / xamstvo	byl žestokim / byla žestokoj / bylo žestokim	daže dlja byvalyx soldat.
punishment$_N$ for deceit$_M$ / vileness$_F$ / rudeness$_N$	was rough$_{M/F/N}$	even for experienced soldiers.
Napadenie na gorod / krepost' / selo	byl soveršen / byla soveršena / bylo soveršeno	v gluxoj predrassvetnyj čas.
attack$_N$ on town$_M$ / fortress$_F$ / village$_N$	was carried.out$_{M/F/N}$	in the early morning.
Ozloblenie na kollektiv / vlast' / gosudarstvo	byl vygodnym / byla vygodnoj / bylo vygodnym	dlja etogo ciničnogo kar'erista.
exasperation$_N$ with co-workers$_M$ / authorities$_F$ / state$_N$	was advantageous$_{M/F/N}$	for this cynical careerist.
Okno v ogorod / step' / pole	byl otkryt / byla otkryta / bylo otkryto	iz-za stojavšej vnutri duxoty.
window$_N$ to garden$_M$ / steppe$_F$ / field$_N$	was opened$_{M/F/N}$	because of the air inside being stuffy.
Opozdanie na samolet / pristan' / sudno	byl neizbežen / byla neizbežna / bylo neizbežnym	iz-za remonta kol'cevoj dorogi.
being.late$_N$ to plane$_M$ / pier$_F$ / ship$_N$	was inevitable$_{M/F/N}$	because of the repairs of the road.
Pis'mo pro poxod / neprijatnost' / priključenie	byl otpravlen / byla otpravlena / bylo otpravleno	iz gluxoj sibirskoj derevuški.
letter$_N$ about camping.trip$_M$ / issue$_F$ / adventure$_N$	was sent$_{M/F/N}$	from a remote village in Syberia.
Pokryvalo na divan / krovat' / kreslo	byl rasšit / byla rasšita / bylo rasšito	v roskošnom vostočnom stile.
cover$_N$ for couch$_M$ / bed$_F$ / arm-chair$_N$	was embroidered$_{M/F/N}$	in splendid oriental style.
Popadanie v stend / mišen' / jabločko	byl vypolnen / byla vypolnena / bylo vypolneno	s nevidannoj prežde točnost'ju.
hitting$_N$ at stand$_M$ / target$_F$ / bull's.eye$_N$	was carried.out$_{M/F/N}$	with an unheard-of accuracy.
Pravo na vybor / pomošč' / sčast'e	byl opredelen / byla opredelena / bylo opredeleno	kak naše neotemlemoe dostojanie.
right$_N$ for choice$_M$ / help$_F$ / happiness$_N$	was defined$_{M/F/N}$	as out inalienable property.

Table 4: Experiment 3 stimuli

Preamble	Predicate	Sent.continuation
Ranenie v život / grud' / plečo<br>wound$_N$ in belly$_M$ / chest$_F$ / shoulder$_N$	byl polučen / byla polučena / bylo polučeno<br>was received$_{M/F/N}$	v xode krovoprolitnogo sraženija.<br>during a slaughterous combat.
Sraženie za aerodrom / krepost' / ukreplenie<br>battle$_N$ for airfiled$_M$ / fortress$_F$ / fortification$_N$	byl perelomnym / byla perelomnoj / bylo perelomnym<br>was critical$_{M/F/N}$	dlja xoda graždanskoj vojny.<br>for the course of the civil war.
Upražnenie na sčet / pamjat' / čtenie<br>exercise$_N$ for counting$_M$ / memory$_F$ / reading$_N$	byl sostavlen / byla sostavlena / bylo sostavleno<br>was developped$_{M/F/N}$	s učetom vozrastnyx osobennostej.<br>with age-specific characteristics in mind.
Allergija na porošok / maz' / lekarstvo<br>allergy$_F$ for powder$_M$ / drug$_F$ / ointment$_N$	byl neožidannym / byla neožidannoj / bylo neožidannym<br>was unexpected$_{M/F/N}$	dlja neopytnogo lečaščego vrača.<br>for the unexperienced physician.
Banka pod gorox / fasol' / varen'e<br>jar$_F$ for peas$_M$ / jam$_F$ / beans$_N$	byl zapolnen / byla zapolnena / bylo zapolneno<br>was filled$_{M/F/N}$	počti do samyx kraev.<br>almost to the rim.
Bor'ba za prestol / vlast' / gospodstvo<br>struggle$_F$ for throne$_M$ / domination$_F$ / power$_N$	byl razvjazan / byla razvjazana / bylo razvjazano<br>was unleashed$_{M/F/N}$	posle neudačnogo dvorcovogo perevorota<br>after an unsuccessful coup d'etat.
Vera v uspex / ljubov' / dobro<br>faith$_F$ in success$_M$ / good$_F$ / love$_N$	byl osnovopolagajuščim / byla osnovopolagajuščej / bylo osnovopolagajuščim<br>was fundamental$_{M/F/N}$	dlja posledovatelej etogo učenija<br>for the followers of this spiritual school.
Dver' v magazin / mečet' / zdanie<br>door$_F$ to shop$_M$ / building$_F$ / mosque$_N$	byl sdelan / byla sdelana / bylo sdelano<br>was made$_{M/F/N}$	iz grubyx počernevšix dosok<br>from rough blackened planks.
Doroga čerez poselok / step' / pole<br>road$_F$ through village$_M$ / field$_F$ / steppe$_N$	byl zabrošen / byla zabrošena / bylo zabrošeno<br>was abandonned$_{M/F/N}$	v konce prošlogo veka.<br>in the end of the last century.
Žaloba na kašel' / migren' / zdorov'e<br>complaint$_F$ about cough$_M$ / health$_F$ / migraine$_N$	byl vosprinjat / byla vosprinjata / bylo vosprinjato<br>was taken$_{M/F/N}$	so vsem vozmožnym vnimaniem<br>with all possible attention.

Table 4: Experiment 3 stimuli

**Preamble**	**Predicate**	**Sent.continuation**
Kniga pro zagovor / junost' / dectvo	byl opublikovan / byla opublikovana / bylo opublikovano	v nebol'šom moskovskom izdatel'stve.
book$_F$ about conspiracy$_M$ / childhood$_F$ / youth$_N$	was published$_{M/F/N}$	in a small publishing house in Moscow.
Nagrada za podvig / smelost' / kačestvo	byl vručen / byla vručena / bylo vručeno	v xode toržestvennoj ceremonii
decoration$_F$ for feat$_M$ / quality$_F$ / courage$_N$	was handed$_{M/F/N}$	at an official ceremony.
Nadežda na uspex / pomošč' / spasenie	byl pročnym / byla pročnoj / bylo pročnym	do samogo poslednego momenta.
hope$_F$ for success$_M$ / rescue$_F$ / help$_N$	was strong$_{M/F/N}$	till the very last moment.
Pereprava čerez potok / propast' / boloto	byl nenadežnym / byla nenadežnoj / bylo nenadežnym	posle nedavnix prolivnyx doždej
bridge$_F$ over torrent$_M$ / swampp$_F$ / precipice$_N$	was unstable$_{M/F/N}$	after recent heavy rains.
Pesnja pro večer / grust' / utro	byl populjaren / byla populjarna / bylo populjarno	sredi prostogo derevenskogo ljuda.
song$_F$ about night$_M$ / morning$_F$ / sadness$_N$	was popular$_{M/F/N}$	among simple villagers.
Provoloka na zabor / izgorod' / ograždenie	byl namotan / byla namotana / bylo namotano	na bol'šuju derevjannuju katušku.
wire$_F$ for fence$_M$ / fence$_F$ / fence$_N$	was reeled$_{M/F/N}$	on a big wooden bobbine.
Rasplata za obman / glupost' / p'janstvo	byl bystrym / byla bystroj / bylo bystrym	iz-za surovosti novogo direktora.
retribution$_F$ for deceit$_M$ / drinking$_F$ / foolishness$_N$	was quick$_{M/F/N}$	because of the rough temper of the new director.
Reakcija na uprek / novost' / soobščenie	byl predskazuemym / byla predskazuemoj / bylo predskazuemym	iz-za carivšego vokrug unynija
reaction$_F$ at reproach$_M$ / message$_F$ / news$_N$	was predictable$_{M/F/N}$	since everybody around was feeling down.
Recenzija na roman / povest' / esse	byl napisan / byla napisana / bylo napisano	dlja izvestnogo literaturnogo žurnala
review$_F$ on novel$_M$ / essay$_F$ / story$_N$	was written$_{M/F/N}$	for a famous literary magazine.
Stipendija za dekabr' / osen' / leto	byl vyplačen / byla vyplačena / bylo vyplačeno	s počti godovym opozdaniem.
stipend$_F$ for December$_M$ / summer$_F$ / autumn$_N$	was pais$_{M/F/N}$	with almost a year of delay.

Table 4: Experiment 3 stimuli

**Preamble**	**Predicate**	**Sent.continuation**
Cena na benzin / neft’ / toplivo	byl povyšen / byla povyšena / bylo povyšeno	po pričine istoščenija zapasov
price$_F$ for gas$_M$ / fuel$_F$ / petrolium$_N$	was raised$_{M/F/N}$	because of the exhaustion of the supplies.
